# IL-9 Producing Tumor-Infiltrating Lymphocytes and Treg Subsets Drive Immune Escape of Tumor Cells in Non-Small Cell Lung Cancer

**DOI:** 10.3389/fimmu.2022.859738

**Published:** 2022-04-20

**Authors:** Lisanne Heim, Zuqin Yang, Patrick Tausche, Katja Hohenberger, Mircea T. Chiriac, Julia Koelle, Carol-Immanuel Geppert, Katerina Kachler, Sarah Miksch, Anna Graser, Juliane Friedrich, Rakshin Kharwadkar, Ralf J. Rieker, Denis I. Trufa, Horia Sirbu, Markus F. Neurath, Mark H. Kaplan, Susetta Finotto

**Affiliations:** ^1^ Department of Molecular Pneumology, Friedrich-Alexander-Universität Erlangen-Nürnberg (FAU), Erlangen, Germany; ^2^ Department of Internal Medicine 1, Friedrich-Alexander-Universität Erlangen-Nürnberg (FAU), Erlangen, Germany; ^3^ Institute of Pathology, Friedrich-Alexander-Universität Erlangen-Nürnberg (FAU), Erlangen, Germany; ^4^ Department of Microbiology and Immunology, Indiana University School of Medicine, Indianapolis, IN, United States; ^5^ Department of Thoracic Surgery, Friedrich-Alexander-Universität Erlangen-Nürnberg (FAU), Erlangen, Germany

**Keywords:** IL-9, immune escape, NSCLC, TIL, tumor immunotherapy

## Abstract

Although lung cancer is the leading cause of cancer deaths worldwide, the mechanisms how lung cancer cells evade the immune system remain incompletely understood. Here, we discovered IL-9-dependent signaling mechanisms that drive immune evasion in non-small cell lung cancer (NSCLC). We found increased IL-9 and IL-21 production by T cells in the tumoral region of the lung of patients with NSCLC, suggesting the presence of Th9 cells in the lung tumor microenvironment. Moreover, we noted IL-9 producing Tregs in NSCLC. IL-9 target cells in NSCLC consisted of IL-9R+ tumor cells and tumor-infiltrating lymphocytes. In two murine experimental models of NSCLC, and *in vitro*, IL-9 prevented cell death and controlled growth of lung adenocarcinoma cells. Targeted deletion of IL-9 resulted in successful lung tumor rejection *in vivo* associated with an induction of IL-21 and reduction of Treg cells. Finally, anti-IL-9 antibody immunotherapy resulted in suppression of tumor development even in established experimental NSCLC and was associated with reduced IL-10 production in the lung. In conclusion, our findings indicate that IL-9 drives immune escape of lung tumor cells *via* effects on tumor cell survival and tumor infiltrating T cells. Thus, strategies blocking IL-9 emerge as a new approach for clinical therapy of lung cancer.

## Introduction

Lung cancer (small and non-small cell lung cancer) represents the leading cause of cancer deaths worldwide (1, 2). In non-small cell lung cancer (NSCLC), growth and progress of the disease is associated with a local expansion of regulatory T cells (Treg) that suppress anti-tumor immune responses thus creating an immunosuppressive environment facilitating immune escape of tumor cells. Recent studies suggest that immunotherapy with PD1/PDL1 checkpoint inhibitors could be a promising approach to activate anti-tumor immune responses and to improve the prognosis for this disease (3, 4). However, the role of cytokines in controlling anti-tumor immune responses in NSCLC remains incompletely understood (5). This aspect is relevant since only 20% of the patients with NSCLC respond to current immunotherapies (6).

Interleukin-9 (IL-9) is a cytokine with pleiotropic functions that was first purified and characterized as a T cell and mast cell growth factor (7). T lymphocytes have been identified as a major source of IL-9 (8–11). However, different T cell subsets, mast cells and innate lymphoid cells-type 2 (ILC2) have the common capability to produce this cytokine (8, 12). Regarding the development of T cells producing IL-9 (Th9) it is known that naïve T-cells in the presence of TGF-beta and interleukin-4 (IL-4) produce IL-9 (13). These cytokines also induce IL-9 production in activated T cells. Hereby, IL-4 activates intracellularly several transcription factors like signal transducer and activator of transcription 6 (STAT6), interferon regulatory factor 4 (IRF4), GATA-binding protein 3 (GATA3), basic leucine zipper transcription factor ATF-like (BATF) and nuclear factor of activated T cells (NFAT) (13, 14). Moreover, the transcription factor PU1 binds to a purine-rich sequence known as the PU-box found on enhancers of target genes. PU1 is a transcription factor of the Erythroblast Transformation Specific (ETS)- family of transcription factors, activated downstream of Transforming growth factor beta (TGF-beta) receptor signaling, that has been demonstrated to bind to the IL-9 promoter in T cells and induces IL-9 production after TGF-beta and IL-4 stimulation in cell culture (11, 15). In addition, several other transcription factors seem to play an important role in the development of Th9 cells including the SMA (“small” worm phenotype) and MAD family (“Mothers Against Decapentaplegic”) of genes known as SMAD proteins as well as the members of the signal transducers and activators of transcription family STAT5 and STAT6 (16). STAT5 is activated downstream of IL-2 and IL-9 signaling and controls IL-9 production in T cells (17). In addition to STAT5, IL-9 activates STAT3 resulting in Th17 and T regulatory cell induction (18). By contrast, STAT6 is the major component of the IL-4-receptor signaling pathway and is therefore also involved in controlling the Th9 phenotype (8). STAT6 is also able to suppress expression of the Th1-associated transcription factor T-box expressed in T cells (T-bet), a known inducer of IFN-ɣ production that inhibits TGFβ-induced expression of Foxp3 (8, 19, 20). It has also been demonstrated that, T cell receptor (TCR) signaling and co-stimulatory molecules regulate IL-9 production. On the one hand, TCR and CD28-mediated co-stimulation leads to the activation of Nuclear Factor of Activated T-cells (NFAT), while on the other hand TCR signaling and OX40 co-stimulation result in activation of nuclear factor-κB (NF-κB) (21).

In agreement with its pleiotropic functions, IL-9 has been demonstrated to influence various different cell types expressing IL-9 receptor (IL-9R) (8, 22, 23). The IL9R consists of 2 subunits: an IL-9R specific alpha chain (IL9R-alpha) and the common gamma chain of the receptor that is shared by other cytokine receptors including receptors for IL-2, IL-4 and IL-7 (23). The IL-9R is expressed on T effector cells rather than in naïve T cells (24). Furthermore, IL-9R is expressed on airway epithelial cells and smooth muscle cells suggesting that several target cell populations for IL-9 exist in lung tissue.

In this study, we determined the expression and function of IL-9 in NSCLC. We demonstrate that IL-9 expression is augmented in NSCLC and plays an important functional role in regulating tumor cell growth. Our findings suggest new avenues for tailored immunotherapy in NSCLC.

## Materials And Methods

### Human Study

Our human study was performed at the Friedrich-Alexander-University Erlangen-Nürnberg, Germany, after being approved by the ethics review board of the University of Erlangen (Re-No: 56-12B; DRKS-ID: DRKS00005376). To date more than one hundred and seventy (170) patients that suffered from primary NSCLC and three metastatic patients underwent surgery and gave their approval to be enrolled in this study in an informed written consent. The patient studies were conducted in accordance with the ethical guidelines of the Declaration of Helsinki. Patients enrolled in this study did not receive any therapy. The confidentiality of the patients was maintained.

Lung cancer diagnosis was based on pathological confirmation. The histological types of lung cancer were classified according to the World Health Organization (WHO) in 2004. Staging was based on the Cancer TNM Staging Manual formulated by the International Association for the Study of Lung Cancer (IASLC), issued in 2010. Clinical data including histological classification, TNM stage, age, gender and smoking status were provided by the Department of Thoracic Surgery and the Institute of Pathology and are summarized in **Table S1**. Clinical data of the control cohort are shown in **Tables S2–S4**.

Immediately after surgery, lung tissue samples were taken from three different regions: the tumoral region (TU: solid tumor tissue), peri-tumoral region (PT: 2-3 cm away from the solid tumor) and the tumor-free control region (CTR: > 5 cm away from the solid tumor). The post-surgery tissue samples were used for RNA and protein isolation as well as for total cell isolation followed by cell culture and FACS analysis. Paraffin-embedded lung tissue arrays were generated as previously described (25) and applied for immunohistochemistry (IHC).

### Antibodies

Antibodies used in this study are listed in the **Supplementary Material**.

### Immunohistochemistry (IHC)

Immunohistochemistry was performed on paraffin-embedded histological sections. Before staining, paraffin was removed from the slides by incubation at 72°C for 30 min and treatment with Roti-Histol (Carl Roth) two times for 10 min. The tissue sections were then rehydrated by immersion in ethanol-series in descending concentrations (100%, 95%, 70%) for 3 min each and in deionized water for 1 min, followed by blocking endogeneous peroxidase in 3% H_2_O_2_ (in methanol) for 20 min. For heat-induced antigen retrieval, slides were placed into a rack containing 50 ml 1 mM Tris-EDTA buffer which was transferred into a pressure cooker followed by incubation at 120°C for 5 min. Slides were then cooled down for 30 min at room temperature (RT) followed by incubation for 1 min in deionized water. Tissue was surrounded with a hydrophobic barrier using a barrier pen. In a next step, slides were stained with the respective primary antibody against CD3, Foxp3, IL-9 or IL-9R **(** **Table S5** **)** using the ZytoChem-Plus AP Polymer-Kit (Zytomed Systems GmbH) according to the manufactures instructions. For IHC single stainings, nuclei were stained with hematoxylin solution (Carl Roth) and slides were covered with coverslips using Aquatex (Merck). For IHC double staining, the second antibody against IL-9 (**Table S5**) was applied and detected according to the manufacture instructions of the Dako EnVision Detection System Kit (Dako Deutschland GmbH). Negative controls were not treated with the primary antibody; the other steps remained the same. Stained slides were scanned using the digital slide scanner (Scan 150) at the Institute of Pathology. Whole slide images were visualized by the CaseViewer software (Version 2.0, 3D Histech Ltd). IL-9 single staining was quantified using the Definiens Tissue Studio 4.1 software (Definiens) while the IL-9R single and the IL-9/Foxp3 double staining have been evaluated using the ImageJ Cell Counter (Version 1.46).

### Cell Isolation and Culture From Lung Tissue

Human tissue samples were cut into small pieces (1-3 mm^2^) using scalpels and digested with Collagenase (2700 U/ml, Sigma-Aldrich: Collagenase from Clostridium histolyticum, Cat# C98991-500MG) and 150µl DNase (10 mg/ml, Roche Diagnostics GmbH: DNase I, Cat#10104159001) diluted in 10 ml R10 medium (500 ml RPMI1640, anprotec, Cat# AC-LM-0060; 50 ml heat-inactivated fetal bovine serum (FCS), Sigma-Aldrich, Cat# S0615; 2mM L-Glutamine, anprotec, Cat# AC-AS-0001; 5 ml Penicillin-Streptomycin (Pen/Strep), anprotec, Cat# AC-AB-0024) in a shaker at 37°C for 1 h. After incubation, the samples were passed through a cell-strainer (100 µm, Greiner Bio-One GmbH: EASYstrainer; Cat# 542000), single-cell suspensions were washed with RPMI1640 and centrifuged at 300g for 7 min at 4°C. The supernatant was removed, and red blood cells (RBC) were lysed in ACK lysis buffer (0,15M NH4Cl, Carl Roth GmbH + Co. KG, Cat# P726.2; 0,01M KHCO3, Carl Roth GmbH + Co. KG, Cat#P748.1; 100M Na2EDTA, GERBU Biotechnik GmbH, Cat# 1034.1000; dissolved and steril filtered in deionized H2O; pH=7.2-7.4) with subsequent centrifugation at 1500rpm for 5 min at 4°C. The cell pellets were washed with Washing Buffer I (500 ml PBS EDTA pH 7.5, BioWhittaker, Cat# BE02-017F; 5 ml Pen/Strep), centrifuged again and resuspended in Washing Buffer II (500 ml PBS EDTA pH 7.5; 5 ml Pen/Strep; 25 ml FCS). Cells were centrifuged at 800rpm for 15 min at 4°C with a low deceleration mode 1. Supernatant was discarded and cells were taken up in Tumor tissue medium (500 ml RPMI1640; 50 ml FCS; 2mM L-Glutamine; 5 ml Pen/Strep; 1mM Sodium-Pyruvate, anprotec, Cat# 11360-070; 5 ml Non-Essential Amino Acids, Gibco, Cat# 11140-035). Cell numbers were determined using Trypan-blue staining in a Neubauer counting chamber. Cells were cultured in Tumor tissue medium at 37°C and 5% CO2.

### Isolation of Peripheral Blood Mononuclear Cells (PBMCs)

PBMCs were freshly isolated using LeucoSep Tubes (Greiner Bio-One, Cat# 227290) according to the manufacturer´s protocol. After cell counting with Trypan-blue in a Neubauer counting chamber, PBMCs were cultured for 4-5 days with 5x10^5^ cells per condition as shown in **Figure 2E**.

### Experimental Skewing Conditions for Foxp3+Treg and IL-9 Producing T Cells in PBMCs

Freshly isolated PBMCs from NSCLC patients and healthy control subjects were cultured in 1ml R10 medium at 5 x 10^5^ cells/well for 4-5 days with plate bound anti-CD3 (1µg/well) and soluble anti-CD28 antibodies (10µg/ml) in a 48 well cell culture plate (Greiner Bio-One, Cat# 677180) at 37°C and 5% CO_2_ (**Figure 2E**). For skewing of IL-9 producing T cells, TGFβ (20ng/ml) and IL-4 (20ng/ml) were added, while the Treg skewing condition included TGFβ (20ng/ml) and IL-2 (2ng/ml). The respective cytokine information are listed in the Table below:

**Table:** 

Cytokine/Antibody	Company	Catalog number
aCD3	BD Biosciences	Cat# 555329
aCD28	BD Biosciences	Cat# 555725
rhIL-2	ImmunoTools	Cat# 11340025
rhIL-4	ImmunoTools	Cat# 11340043
hTGFβ	Miltenyi Biotec	Cat# 130-095-067

### Flow Cytometric Analysis

To prevent unspecific binding of antibodies to Fc receptors, single cell suspensions were incubated with unlabeled antibodies against IgG Fc receptors CD16/32 (human: 1:100, BD Biosciences Cat# 564220; murine: 1:100, BD Biosciences Cat# 553142) in FACS Buffer (500 ml PBS, anprotec Cat# AC-BS-0002; 10 ml FCS) for 5 min at 4°C. Cells were washed with FACS Buffer and centrifuged. Pellets were resuspended with FACS Buffer containing antigen-specific fluorochrome-conjugated antibodies in appropriate concentrations and incubated for 15 minutes at 4°C in the dark. After cells were washed with FACS Buffer and centrifuged, pellets were either resuspended in FACS Buffer to measure directly the surface stained cells or proceeded to intracellular staining. Therefore, the FoxP3 staining kit (Thermo Fisher Scientific Cat# 00-5523-00) was applied according to the manufacturer’s protocol. Permeabilized cells were stained for intracellular proteins in a volume of 50 µl PermWash Buffer supplemented with antibodies in appropriate concentrations. Cells were washed, taken up in FACS Buffer and analyzed immediately **(** **Table S6** **).** Flow cytometric analyses were obtained with the FACS Canto II and the BD FACS Diva software (both BD Biosciences) for compensation. Data were analyzed with Flow-Jo v10.2 (FlowJo) software always gating for singlets using the forward scatter and for living cells using forward scatter vs. sideward scatter before specific analysis (also shown in **Supplementary Material**).

Cells were first stained with Zombie (1:500, Biolegend, Cat#423101/423102) for 15 min at room temperature prior to Fc blocking and washed twice with FACS buffer. The staining of surface proteins was then continued as described above.

Data of applied FMOs (Fluorescence minus one) are shown in **Supplementary Material**.

### RNA Isolation and cDNA Synthesis

Lung tissue samples were homogenized using Precellys Lysing Kits (Bertin Technologies, Cat# P000918-LYSK0-A) and the benchtop homogenizer Minilys (Bertin Technologies) as described in the manufacturer’s protocol. RNA of homogenized samples or single cell suspensions was isolated using peqGold RNA Pure (Peqlab, Cat# 30-1010) or Qiazol Lysis Reagent (Qiagen, Cat# 79306) according to the manufacturer´s instructions. RNA was reversely transcribed into cDNA using the RevertAid First Strand cDNA Synthesis Kit (ThermoFisher Scientific, Cat# K1622) according to the manufacturer’s protocol.

### Quantitative Real-Time PCR (qPCR)

qPCR of synthesized cDNA was performed by using iTaq Universal SYBR Green Supermix (Bio-Rad Laboratories, Cat# 1725124) in a total volume of 20 µl. Primer sequences for murine and human qPCR analyzes are depicted in **Supplementary Table S7** which were purchased from Eurofins-Genomics Germany. qPCR Reactions (50 cycles, initial activation 98°C, 2 min, denaturation 95°C, 5 min, hybridization/elongation 60°C, 10 min) were performed using the CFX-96 Real-Time PCR Detection System and analyzed by the respective CFX Manager Software (both Bio-Rad Laboratories). Data were analyzed using the relative quantification 2^-ΔΔCT^ method by normalization to the housekeeping-gene hypoxanthine-guanine-phosphoribosyltransferase (Hprt).

### ELISA

The enzyme-linked immunosorbent assay (ELISA) technique was utilized to analyze the cytokine concentration in cell culture supernatants, BALF and Serum. ELISA was performed in accordance with the manufacturer’s instructions. Human ELISA Sets were purchased as followed: IL-9 ELISA (Human IL-9 DuoSet ELISA, R&D Systems, Cat# DY209-05), IL-21 ELISA (Human IL-21 DuoSet ELISA, R&D Systems, Cat# DY8879-05), IFNγ ELISA (BD OptEIA™ Human IFN-γ ELISA Set, BD Biosciences, Cat# 555142). Murine samples were analyzed by: IL-21 ELISA (Mouse IL-21 DuoSet ELISA, R&D Systems, Cat# DY594), IFNγ ELISA (BD OptEIA™ Mouse IFNγ ELISA Set, BD Biosciences, Cat# 555138), IL-10 ELISA (BD OptEIA™ Mouse IL-10 ELISA Set, BD Biosciences, Cat# 555252).

### Protein Extraction and Western Blot Analysis

Lung tissue samples from patients were lysed with RIPA Buffer (Thermo Fisher Scientific, Cat# 89900) and inhibitor cocktail (complete Mini EDTA-free, Roche Diagnostics, Cat# 11836170 001) then homogenized with SpeedMill PLUS (Analytik Jena) in innuSPEED lysis Tubes P (Analytik Jena, Cat# 845-CS-1020250) and centrifuged (3000rpm, 5min, 4°C). The supernatant was incubated for 30 minutes on ice and centrifuged again (2000g, 5min, 4°C). Final extraction was done by another centrifugation step of the supernatant (45min, maximum speed, 4°C) and measured with Bradford Assay (Protein Assay Dye Reagent Concentrate, Bio-Rad, Cat# 5000006), followed by protein denaturation (95°C, 5 min) in a mix of reducing loading buffer (4xLDS Sample Buffer, Thermo Fisher Scientific, Cat# NP0007) and DTT (1M, Thermo Fisher Scientific, Cat# P2325). A mini-PROTEAN TGX Stain-Free Gel (Bio- Rad, Cat# 4568086) was used with 20µg of proteins per well and at 120V, 150mA for 1h. Proteins were then transferred to a 0,2µm nitrocellulose membrane (Trans-Blot Turbo Transfer Pack, Bio-Rad, Cat# 1704158) by using a western blot trans-blot turbo system (Bio-Rad). Transfer and protein load was assessed and recorded by using a ChemiDoc Imaging System (Bio-Rad). After washing and blocking (3% powdered milk (Carl Roth GmbH, Cat# T145.3) in Tween SDS buffer) the membrane for 1h at room temperature, the primary antibody was applied and incubated over night at 4°C. After that, the membrane was washed and incubated with the compatible secondary antibody in blocking buffer, for 1h at room temperature. Respective antibody information are listed in **Table S8**. After final washing steps, detection was performed using SuperSignal™ West Femto Maximum Sensitivity Substrate (Thermo Scientific, Cat# 34095). Visualization of the western blot results was done by using the ChemiDoc Imaging System. Protein bands and total protein levels were analysed with the ImageLab software (Bio-Rad).

### Cell Lines

The human A549 (ATCC^®^ CCL-185™) cell line was purchased authenticated from the ATCC bank (Manassas, Virginia, USA). Cells were cultured in F-12K Nut mix medium (Thermo Fisher Scientific, Cat# 21127-022) supplemented with 10% FCS, 1% Pen/Strep and 1% L-Glu at 37°C and 5% of CO_2_.

The murine LL/2-luc-M38 (LL/2) cell line was purchased and authenticated from Caliper LifeScience (Bioware cell line, Caliper LifeScience, Waltham, Massachussets, USA). All Caliper Life Sciences cell lines are confirmed to be pathogen-free by the IMPACT profile I (PCR) at the University of Missouri Research Animal Diagnostic and Investigative Laboratory. LL/2 cells were cultured in D10 medium (anprotec, Cat# AC-LM-0012; 50 ml heat-inactivated fetal bovine serum (FCS), Sigma-Aldrich, Cat# S0615; 2mM L-Glutamine, anprotec, Cat# AC-AS-0001; 5 ml Penicillin-Streptomycin (Pen/Strep), anprotec, Cat# AC-AB-0024) at 37°C and 5% of CO_2_.

The murine bronchoalveolar carcinoma cell line L1C2 (Dubinett et al. https://pubmed.ncbi.nlm.nih.gov/8382559/) was obtained from Prof R. Wiewrodt. Cells were cultured in R10 Medium at 37°C and 5% CO2.

### Cell Line Experiments

3x10^5^ A549 were cultured overnight in 6-well plates (Greiner Bio-One, Cat#657160) in supplemented F-12K Nut mix or R10 medium with or without 50 ng/ml IL-9 (ImmunoTools, Cat# 11340093) for 72h. Other different experimental conditions were described in the respective figure. For the Fas agonistic challenge, we incubated A549 with 2 μg/mL anti-CD95 (Biolegend, Clone DX2, Cat# 305655) or IgG1, k (Biolegend, Clone MOPC-21, Cat# 400153).

1.25x10^5^ LL/2 cells were cultured in 24-well cell culture plates (Greiner Bio-One, Cat# 662160) for 24h at 37°C and 5% CO2 in D10 medium with 30 ng/ml or 100 ng/ml IL-9 or left untreated. Cells were harvested and counted for live and dead cells using trypan blue staining.

5x10^5^ LL/2 or L1C2 cells were cultured overnight in 6-well plates in D10 or R10 medium, respectively. After the first 24h the cells were stimulated with 30 ng/ml or 100 ng/ml IL-9 or left untreated for another 24h.

### Apoptosis Analysis by Flow Cytometry

Determination of apoptotic cells was performed using Annexin V/PI staining according to the manufacturer’s protocol (BD Bioscience; Cat#550474 and #556463). Briefly, 1x10^5^ cells were stained with 5 μl AnnexinV-APC and 5 μl PI in 100 μl 1x AnnexinV binding buffer for a total volume of 110 μl and incubated for 15 min at RT in the dark. Reaction was stopped by adding 200 μl 1x AnnexinV binding buffer and samples were immediately analyzed with the FACSCanto II. Data sets were evaluated by Flow-Jo v10.2.

### Proliferation Analysis by Flow Cytometry

Determination of proliferating cells was performed using Ki67 staining according to the manufacturer’s protocol (BD Bioscience, Cat# 556026). For Ki67 analysis, 1x10^6^ LL/2 or L1C2 cells were fixed with 75% ethanol and incubated for at least 2 hours at -20°C. After washing the cells with FACS buffer the cells were stained with 20 µl Ki67-FITC diluted in FACS buffer for a total volume of 120 µl and incubated for 30 min at RT in the dark. Reaction was stopped by adding 1 ml PBS, centrifuged and resuspended in 250 µl PBS. Cells were immediately analyzed using the FACSCanto II. Data sets were evaluated by Flow-Jo v10.2.

### Experimental Mice Models

All mice were bred and maintained under specific-pathogen-free (SPF) conditions in our animal facility (University Hospital Erlangen, Hartmannstraße 14, Erlangen). All experiments were performed in accordance with the German and European laws for animal protection and were approved by the local ethics committees of the Regierung Unterfranken (Az 55.2-2532-2-1286-20). IL-9^-/-^ mice were bred on Balb/c and C57BL/6 genetic backgrounds. And they were kindly presented by PD Dr. Benno Weigmann and PD Dr. med. Andreas Ramming from University Hospital Erlangen Medicine 1 and Medicine 3, respectively (26).

### LL/2 Induced Lung Adenocarcinoma Model and Antibody Treatment

For lung tumor induction, 5x10^5^ cells resuspended in 200 µl DMEM medium (without supplements) were injected into the tail vein of 6-8 weeks old female mice. At the indicated time points, mice were weighed and injected intraperitoneally (i.p.) with luciferin (0.15 mg per 1 g body weight; Promega, Cat#P1043). Luciferase activity was measured after 20 min by the IVIS Spectrum *In Vivo* Imaging System (PerkinElmer) as previously described (5). Briefly, mice were anaesthetized using isoflurane and luciferase activity was measured by detecting luminescence intensity (photons per second). Analyses were performed in a logarithmic scale mode. Mice were sacrificed at day 14-23 after tumor cell injection. For the inhibition of IL-9 *in vivo*, we used two different protocols. In the first protocol, mice were treated i.p. with 200 µg of anti-IL9 antibody (BioXCell, Clone 9C1, Cat# BE0181) or IgG2a Isotype control (BioXCell, Clone C1.18.4, Cat# BE0085) dissolved in 100 µl PBS at day 1, 3, 6, 9, 13 and 16 after tumor induction with an experiment termination at day 21 to 23 depending on the lung tumor load. In the second protocol, mice were treated i.p. with 20 µg anti-IL9 antibody or IgG2a Isotype control resolved in 200 µl PBS at day 6, 9, 10 and 13 after tumor induction with an experiment termination at day 20.

### L1C2 Induced Lung Adenocarcinoma Model

For lung tumor induction by L1C2, 2x10^5^ cells resuspended in 200 µl DMEM or RPMI medium (without supplements) were injected into the tail vein of 6-8 weeks old female mice. Mice were sacrificed at day 14-23 after tumor cell injection, as indicated.

### Hematoxylin and Eosin (H&E) Staining on Murine Paraffin-Embedded Lung Sections and Analysis of Tumor Load

Representative parts of the sectioned mouse lung were fixed in 10% formalin-PBS solution, dehydrated, and embedded in paraffin. Five-micrometer-thick lung sections cut from paraffin blocks were stained with hematoxylin and eosin for visualization of lung tumors. Stained slides were scanned using a digital slide scanner (Scan 150, 3D Histech Ltd) and slide images were visualized using the CaseViewer software (Version 2.0, 3D Histech Ltd). Staining and scanning of slides was performed in cooperation with the Institute of Pathology in Erlangen

To analyze the tumor load in the lung for each mouse, the lung sections were histologically evaluated after Hematoxylin and Eosin staining. A pathologist analysed in a blinded codified manner the tumor load of the murine lungs. Moreover, each section was analyzed by means of area [µm²] and number of foci. The sections were investigated under the microscope with an object magnified 10 times (Carl Zeiss, AxioCam MRC) and live pictures were directly transmitted to the Zen 2 blue edition software (Carl Zeiss Microscopy GmbH). With the help of the graphic tool, the foci were localized, circled and automatically measured by means of area. The obtained data corresponding to each mouse was used for comparisons as well as correlation analyzes and transferred into graphs (GraphPad Prism 8).

### Isolation of Total Murine Lung Cells

Dissected lungs were cut into small pieces using scalpels and digested with Collagenase (2700 U/ml; Sigma-Aldrich: Collagenase from Clostridium histolyticum; Cat# C98991-500MG) and 1,5 mg DNase (10 mg/ml; Roche Diagnostics GmbH: DNase I; Cat#10104159001) diluted in 10 ml R10 medium in a shaker at 37°C for 1 h. After incubation the samples were passed through a cell-strainer (40 µm, Company, Cat#542040), single-cell suspensions were washed with RPMI1640 and centrifuged at 1500rpm for 10 min at 4°C. The supernatant was removed and RBC were lysed in 10 ml ACK lysis buffer with subsequent centrifugation at 1500rpm for 5 min at 4°C. The cell pellets were washed with 10 ml PBS and centrifuged at 1500rpm for 5 min at 4°C. After removal of the supernatant, the cells were taken up in 10 ml R10 medium and cell numbers were determined using Trypan-blue staining in a Neubauer counting chamber.

### Isolation of Murine Lung CD4+ T Cells

CD4+T cells were isolated from the lungs of tumor-bearing mice by magnetic cell separation using anti-CD4 MicroBeads (Miltenyi Biotec) according to the manufactures protocol. The purity of isolated CD4+ T cells was confirmed by FACS analysis. Isolated T cells were then cultured in RPMI medium supplemented with 10% FCS, 1% Pen/Strep and 1% L-Glu at 37°C and 5% CO_2_, in the presence plate-bound anti-CD3 (5 µg/ml, BD Biosciences, Cat#) and soluble anti-CD28 (1 µg/ml, Biolegend, Cat#) antibodies. Supernatants and cells were harvested after 24h of cell culture for further analyses.

### Mouse CD4+ T Cell Isolation and *In Vitro* Differentiation

Naïve CD4 T cells were isolated from mouse spleens using the CD4^+^CD62L^+^ T cell isolation kit according to the manufacturer’s protocol (Miltenyi Biotec). Cells were cultured in R10 medium on anti-CD3 (2 µg/ml; BioXCell) coated cell culture plates with soluble anti-CD28 (2 µg/ml; BioXcell). Cells were cultured under Treg polarizing conditions including hTGF-β1 (2 ng/ml), hIL-2 (50 U/ml), anti-IFN-γ (XMG; 10 mg/ml) and anti-IL-4 (11B11; 10mg/ml). Th9 cells were cultured with hTGF- β1 (2 ng/ml), IL-4 (20 ng/ml), hIL-2 (50 U/ml) and anti-IFN-γ (10 mg/ml). Th0 cells were cultured with hIL-2 (50 U/ml), anti-IFN-γ (XMG; 10 mg/ml) and anti-IL-4 (11B11; 10mg/ml). On day 3, cells were expanded into fresh media containing the original concentrations of cytokines in the absence of co-stimulatory signals for additional 2 days. On day 5, mature T cell subsets were harvested for further analysis.

### Flow Cytometric Analysis of Cultured Treg Cells

For transcription factor staining in Treg cells from different culture conditions were harvested on day 5 of differentiation whereas for cytokine staining, CD4+ T cells were stimulated with Phorbol 12-myristate 13-acetate (PMA, 5ng/ml, Sigma-Aldrich) and ionomycin (500ng/ml, Sigma-Aldrich) for 3 hours followed by monensin (2μM, Biolegend) for total 6 hours at 37°C. Cells were washed with FACS buffer (PBS with 0.5% BSA). CD4+ T cell subsets were then stained with a fixable viability dye (eBioscience) and surface markers (CD4, RM4-4, Biolegend; CD25, PC61, Biolegend) for 30 min at 4°C followed by washing and fixation with 4% formaldehyde for 10 min at room temperature. For transcription factor staining, after surface staining, cells were fixed with Fixation & Permeabilization Buffer (FoxP3 staining kit, Thermo Fisher Scientific, Cat# 00-5523-00) for 2 hours at 4°C, and then permeabilized with permeabilization buffer. Cells were stained with Foxp3 antibody (Foxp3, FJK-16s, eBioscience) for 30 min at 4°C followed by flow-cytometry. For cytokine staining, after fixation cells were then permeabilized with permeabilization buffer, and stained for cytokines (IL-9; RM9A4, Biolegend).

### Gene Expression and IL-9 Analysis in Cultured Treg Cells

Total RNA was isolated from cells using TRIzol (Life Technologies). RNA was reverse transcribed according to manufactures directions (Quantabio, Beverly, MA). Quantitative Reverse Transcriptase (qRT-PCR) was performed with commercially available primers (Life Technologies) with a 7500 Fast-PCR machine (Life Technologies). Gene expression was normalized to housekeeping gene expression (β2-microglobulin).

For measurement of IL-9 secretion, on day 5, 1x10^6^ cells were re-stimulated with anti-CD3 coated on 12 well cell culture (Greiner Bio-One, Cat# 665180) plates for 12 hours. Supernatants were collected and IL-9 secretion was measured using IL-9 ELISA MAX™ kit provided by BioLegend (Cat#442704).

### Th1-*In Vitro* Differentiation

Naïve CD4^+^CD62L^+^ T cells were isolated from the spleens of naïve mice by magnetic cell sorting using the CD4^+^CD62L^+^ T Cell Isolation Kit II (Miltenyi Biotec). The purity of the cell isolation was confirmed by FACS analysis.

For T cell differentiation, CD4^+^CD62L^+^ T cell were cultured in RPMI medium supplemented with 10% FCS, 1% Pen/Strep and 1% L-Glu, at 37 °C and 5 % CO_2_ in the presence of plate-bound anti-CD3 (5 µg/ml, BD Biosciences) and soluble anti-CD28 (1 µg/ml, Biolegend) antibodies.

For Th1 differentiation, cells were additionally treated with 5 µg/ml anti-IL-4 antibody (BioLegend) and 12 ng/ml recombinant IL-12 (PeproTech).

Afterwards ACK lysis buffer was applied as previously described (27).

### Statistical Analysis

All data analysis was done using Prims software version 8 (Graphpad Software) using a Student’s test, ordinary one-way ANOVA or two-way ANOVA. *Post hoc* Tukey test was used for multiple comparisons. Simple linear regression was used for the correlation. P<0.05 was considered statistically significant.

## Results

### Increased IL-9 Production by T Cells in the Tumoral Region of the Lung of Patients With NSCLC

In this study, we aimed for characterizing the expression and function of IL-9, a cytokine with immune-regulatory functions (28, 29), in non-small cell lung cancer (NSCLC).

**Figure 1:**
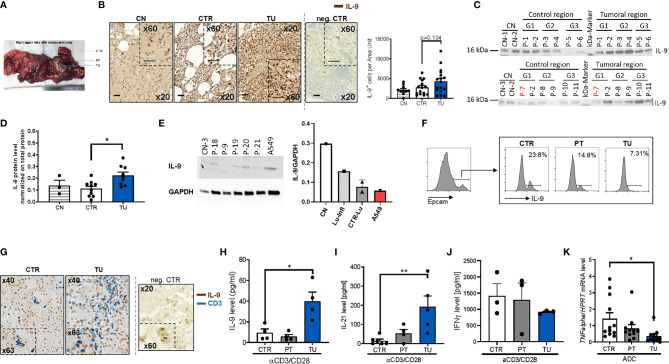
Increased IL-9 production by T cells in the tumoral lung region of NSCLC patients. **(A)** Representative photographic image of the resected lung of one NSCLC patient as a showcase of defined regions implemented for our human study cohort. Lung tissue samples were dissected from the tumoral area (TU), the peri-tumoral area (PT) surrounding the tumor and from the control area (CTR) consisting out of healthy lung tissue. **(B)** IL-9 immunohistochemistry (IHC) on paraffin-embedded tissue arrays obtained from the lung of tumor-free control patients (CN) or the CTR and TU lung regions of non-small cell lung cancer (NSCLC) patients (nCN=10; nCTR=15; nTU=17) (scale bar=50 µm). **(C, D)** Proteins were isolated from lung tissue samples of control patients (CN), the CTR and the TU of NSCLC ADC patients (classified by grading of tumor cell differentiation (G1, G2 and G3) and Western blot was performed. Detected protein levels of IL-9 were normalized on total protein of the samples (CN: n=3, CTR: n=10, TU:n=10). **(E)** Proteins were isolated from tissue samples of control patients that underwent surgery due to disease unrelated to tumor and from cells of the adenocarcinoma cell line A549. Analysed data are shown as mean ± SEM (nCN=1; nLu-Infl (Lung inflammation)=1; CTR-Lu=CTR, n=2; nA549 = 1) or single values. **(F)** Flow cytometry analysis of IL-9+ cells (shown in percentage) gated on Epcam+ cells of cells isolated from the CTR, the PT and the TU of a NSCLC patient. **(G)** Double IHC for IL-9 (brown) and CD3 (blue) on lung tissue obtained from the CTR and TU regions of an adenocarcinoma **(**ADC) patient (scale bar=50 µm**). (H)** ELISA analysis of IL-9 levels (pg/ml), **(I)** IL-21 levels (pg/ml), **(J)** IFNγ (pg/ml) in supernatants obtained from total cells isolated from the control (CTR), peri-tumoral (PT) and tumoral (TU) region of adenocarcinoma (ADC) patients and cultured with anti(α)-CD3/CD28 antibodies for 24h (IL-21: nCTR=6, nPT=4, nTU=5; IFNγ: nCTR=3; nPT=3; nTU=3). **(K)** TNF-alpha mRNA level from total lung cells was normalized on HPRT mRNA level (nCTR=12, nPT=11, nTU=12). N values are given per group. Bar charts indicate mean values +/- s.e.m. using student´s two-tailed t-test *P,0.05; **P,0.01; ***P,0.001.

To investigate the IL-9 expression in the lung of patients with NSCLC, we did immunohistochemistry for IL-9 on post-surgery lung samples collected on tissue arrays and compared IL-9 production from the tumoral region (TU: solid tumor tissue), and the tumor-free control region (CTR: > 5cm away from the solid tumor) in NSCLC (see *Materials and Methods* and **Figure 1A**). The clinical data of these patients as well as the healthy subjects whose post-surgery lung samples were analyzed in this study, are reported in **Tables S1, S2**, respectively. This analysis showed induction of IL-9+ cells in the tumoral region of the lung as compared to the control region in these lung sections derived from patients with NSCLC **(** **Figure 1B** and **Supplementary Figure 1A** **).** Patients included in this study were not treated with any immunotherapy before undergoing surgery analysis. To further analyze the IL-9 induction in the tumoral region of these patients, we next analyzed interleukin 9 expression in proteins extracted from the control and tumoral regions of the lung of NSCLC patients. Furthermore, we analyzed the lung of 3 healthy controls (CN) and one subject with lung inflammation (Lu-infl.) by using Western blot analysis **(** **Figures 1C–E** and **Supplementary Figure 1B** **).** Here, we confirmed upregulation of IL-9 in the tumoral region of the lung of patients with NSCLC **(** **Figures 1C, D** **)**.

We next started to investigate the cellular sources of IL-9 in NSCLC. Since IL-9 was induced in the tumoral region of the lung, we asked whether the tumor cells themselves would produce IL-9. To do so, we cultured the lung tumor adenocarcinoma cell line A549 and extracted proteins for performing Western blot analysis for IL-9. We found that A549 cells produce IL-9 although not as high as control healthy lungs when corrected for GAPDH expression **(** **Figure 1E** **).**

We further investigated IL-9 in epithelial cells isolated from the lung of one NSCLC patient and found decreased numbers of epithelial cells (EpCAM+) producing IL-9 (IL-9+ cells) in the tumoral region as compared to the control region by flow cytometric analysis **(** **Figure 1F** **).** Furthermore, we found few epithelial cells expressing IL-9 distributed in the different regions of the lung of the patients with NSCLC by immunohistochemistry (IHC) **(** **Supplementary Figure 1A** **)**. We next further characterized the cellular source of IL-9 in lung tissue from NSCLC patients and focused our analysis on IL-9 production by tumor infiltrating T cells (TIL) and performed double immunohistochemistry with anti-IL-9 and anti-CD3 antibodies in lung tissue arrays as in **Figure 1B**. An example of this staining is shown in **Figure 1G**. Here, we found double positive cells, indicating the presence of lung CD3+ T cells producing IL-9, which infiltrated the tumoral regions of the lung in NSCLC. Accordingly, we analyzed IL-9 production of total lung cells from the three lung tumor regions after T cell stimulation with anti-CD3/CD28 antibodies in a cohort of NSCLC patients by ELISA **(** **Figure 1H** **)**. Here, we noted significantly augmented IL-9 production by cultured lung cells stimulated with aCD3/28 antibodies from the TU region in NSCLC as compared to the CTR and PT regions of these patients. As IL-9 from T cells is mainly produced by Th9 cells which also produce IL-21 (20, 28), we next determined IL-21 concentration in the same cell supernatants where IL-9 was detected. We found a significant upregulation of IL-21 production in the supernatants of aCD3/aCD28 antibodies cultured total lung cells from the TU region of patients with NSCLC as compared to the CTR region **(** **Figure 1I** **).** This finding indicates higher T cell infiltration as the possible cause for the increased IL-9 levels found in the tumoral area of the lung of NSCLC patients (30). By contrast, the anti-tumoral cytokine IFN-ɣ was found, by trend, downregulated in these lung cells isolated from the tumoral region **(** **Figure 1J** **)**. In confirmation of decreased anti-tumoral cells infiltrating the tumoral region of NSCLC patients, we found significantly less TNF-alpha mRNA levels in mRNA isolated from the lung tumoral regions as compared to the respective control region **(** **Figure 1K** **).**

In conclusion, we found that TIL in the tumoral region of NSCLC patients produced IL-9 while challenged with anti-CD3/CD28 antibodies. Moreover, in addition to tumor infiltrating lymphocytes, also lung tumoral cells have the capacity to produce IL-9.

### Increased IL-9 Production by T Regulatory Cells in the Tumoral Region of the Lung of Patients With NSCLC

**Figure 2:**
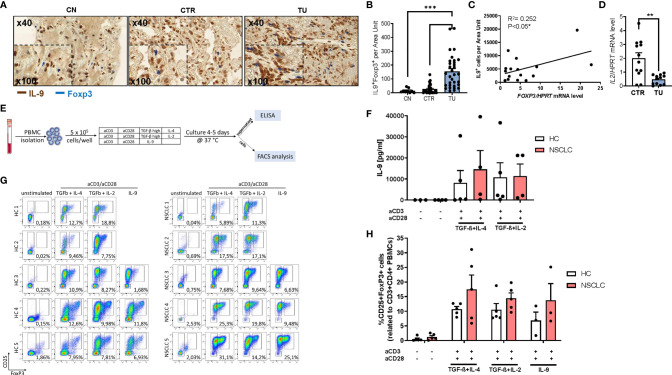
Increased IL-9 production by regulatory T cells (Tregs) of patients with NSCLC. **(A)** Immunohistochemistry staining of lung tissue sections for IL-9 (colored in brown) and Foxp3 (colored in blue). Representative microscopy images are shown for one healthy control patient (CN) and the CTR and TU lung region from one NSCLC patient. Images were taken at a 40x magnification while inserts show a magnification of 100x. **(B)** Total IL-9 and FoxP3 double-positive cells per area are shown for CN (n=10) and NSCLC CTR (n=35) and NSCLC TU (n=34) Data are shown as mean + SEM. using student´s two-tailed t-test *P,0.05; **P,0.01; ***P,0.001. **(C)** Correlation of IL-9^+^ cells and FoxP3 mRNA levels from the tumoral region of NSCLC patients. Correlation was done with IL-9^+^ cells from the TU of immunohistochemistry staining analysis and FoxP3 mRNA levels of the TU from the same patient. mRNA levels were detected by RT-qPCR and set in relation to HPRT mRNA level (n=16). **(D)** qPCR based analysis of IL-2 mRNA levels of CTR and TU set in relation to HPRT mRNA level (nCTR=12; nTU=11). **(E)** Experimental design for the *in vitro* culture of PBMCs. PBMCs from NSCLC patients or healthy control patients were isolated and cultured with different conditions for 4-5 days at 37°C and 5% CO_2_ (500.000 cells/well). After harvesting the cells, the supernatant was used to perform ELISA and the cells were analyzed by flow cytometry. **(F)** Analysis of IL-9 concentration (pg/ml) in the supernatant of PBMC cell culture from healthy controls (n=3-5) and NSCLC patients by ELISA (n=4. **(G)** Representative flow cytometry analysis of CD25^high^FoxP3^+^ cells (%) gated on CD3^+^CD4^+^ lymphocytes (n=5). Representative dot-plots showing CD25 and FoxP3 staining of PBMCs from control patients and NSCLC patients after cell culture with different conditions (unstimulated; IL-4 (20 ng/ml) and TGFβ (20 ng/ml); Treg: IL-2 (2 ng/ml) and TGFβ (20 ng/ml); IL-9 (20 ng/ml)). **(H)** Quantification of CD25^high^FoxP3^+^ Tregs (n_HC_=5, n_NSCLC_=5; IL9-condition: n_HC_=3, n_NSCLC_=3). For statistical analysis One-way ANOVA test was applied. *p < 0.05.

We previously described induced immunosuppression in the tumoral region of the lung of patients with NSCLC (31, 32). To determine whether regulatory T (Treg) cells would produce IL-9 in the lung of patients with NSCLC, we performed double immunohistochemistry for IL-9 and Foxp3 in tissue array slides **(** **Figures 2A, B** **).** Here, we found an increased number of double positive cells for Foxp3 and IL-9 in the tumoral region compared to the control region of the lung of NSCLC patients suggesting that lung Treg cells may produce IL-9 in NSCLC. Consistently, we found a positive correlation between the Foxp3 mRNA levels and IL-9+ T cells in the tumoral area in NSCLC **(** **Figure 2C** **).**

Treg cells are known to be induced by low levels of IL-2, hereby competing with TIL for IL-2 induced survival (33, 34). By having a view on IL-2, we noted reduced IL-2 mRNA expression in the tumoral region compared to the control region **(** **Figure 2D** **),** indicating that low levels of IL-2 and the presence of other immunosuppressive cytokines like TGF-beta and IL-10 in the tumor microenvironment may contribute to augmented Treg numbers in NSCLC (31, 35).

### PBMCs From NSCLC Patients Release Augmented Amounts of IL-9 Upon Specific Skewing Conditions

To determine the capacity of Treg cells to produce IL-9, we isolated peripheral blood mononuclear cells (PBMCs) from five NSCLC patients and five healthy control subjects. Next, the PBMCs were cultured under T cell producing IL-9 and Treg skewing conditions with anti-CD3 and anti-CD28 antibodies. After 4-5 days of cell culture, we performed FACS analysis and measured IL-9 in the supernantant by ELISA (**Figures 2E, F** and **Figure S2A** **)**. In the cell supernatants from PBMCs derived from both healthy controls as well as NSCLC patients, we observed an induction of IL-9 under conditions inducing IL-9 (TGF-beta+IL-4) as well as under immunosuppressive conditions (TGF-beta+ low IL-2) **(** **Figure 2F** **).** Moreover, we could detect induced CD3+CD4+CD25+Foxp3+ T regulatory cells both in Treg and IL-9 inducing conditions in PBMCs from both healthy control subjects and from NSCLC patients (**Figures 2G, H** **).** Thereby the NSCLC group showed an enhanced induction of Treg frequency. These data indicate that factors present in the tumor microenvironment like TGF-beta and low IL-2, can induce IL-9 in TILs from patients with NSCLC and simultaneously induce Foxp3+ T regulatory cells. In addition, our results from the peripheral blood demonstrate that immunosuppression can drive IL-9 both in healthy and in NSCLC subjects.

### Human NSCLC Tumor Cells and TILs Express IL-9R

**Figure 3:**
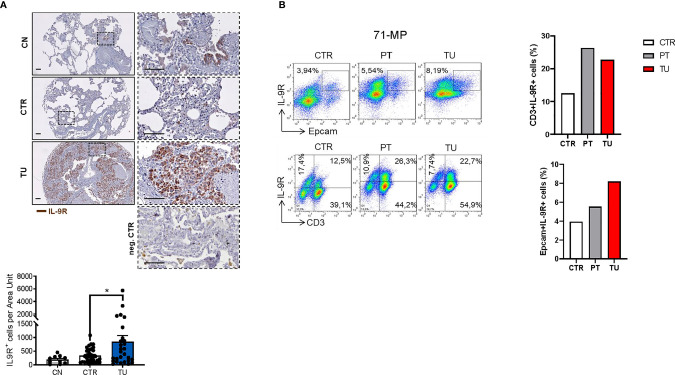
Increased IL-9 Receptor expression in the lung of NSCLC patients. **(A)** Immunohistochemistry staining of lung tissue sections for IL-9R (colored in brown). Representative microscopy images are shown for one healthy control patient (CN), the CTR and TU from one NSCLC patient and a negative Control. Images were taken at a 20x magnification while squared areas are shown below at a magnification of 60x. Quantification of the data is shown below as mean ± SEM using ordinary one-way ANOVA (nCN=10, nCTR=33, nTU=28), *P,0.05; **P,0.01; ***P,0.001. **(B)** Flow cytometry analysis of IL-9R^+^ cells (%) in association with Epcam^+^ and CD3^+^ lung tissue cells derived from the CTR, the PT and the TU from a representative patient with NSCLC (patient ID: 71-MP). Percentage of CD3+IL-9R+ and EpCAM+IL-9R+ cells is shown in the right.

Lung cancer immunosuppression is mediated *via* effects of tumor cells on TILs resulting in T cells expressing Foxp3+ and IL-10 (35). To determine whether this concept is also relevant for tumor cells and TILs in NSCLC patients, we next assessed the distribution of IL-9R in patient samples. To this end, we performed immunohistochemistry for IL9R on the lung tissue arrays of our cohort of NSCLC patients and found an upregulation of IL-9R in the tumoral region as compared to the control region and healthy controls **(** **Figure 3A**).

Flow cytometric analysis of TILs from lungs of NSCLC patients revealed an upregulation of IL-9R expression in the CD3+ T cells localized in the peritumoral and tumoral region of the lung as compared to control regions **(** **Figure 3B**, upper graph). We next addressed IL-9R expression in epithelial cells, as this is developed in lung tumors upon epithelial transformation. We found that IL-9R expression was increased by trend in Epcam+ epithelial cells in the lung tumoral region, as compared to the control and the peri-tumoral regions in patients with lung adenocarcinoma, indicating that normal epithelial cells as well as tumor cells express comparable amounts of IL-9R **(** **Figure 3B** **).**

**Figure 4:**
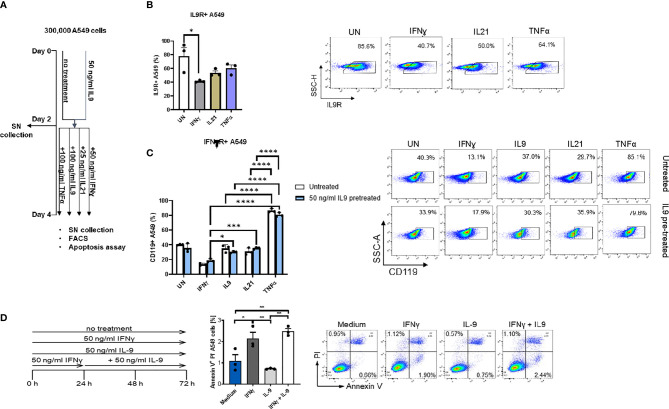
Analysis of IL9 effects on cell surface receptors IL-9R and IFNγ-R expression *in vitro* on A549 cell line. **(A)** Experimental design of A549 cell line culture for 4 days. A549 cells were pretreated with or without IL9 (50 ng/ml) for two days. On day 2, supernatants were collected and cells were further cultured for two days with IFNɣ (50 ng/ml), IL9 (100 ng/ml), IL21 (25 ng/ml) or TNFα (100 ng/ml) in new medium, respectively. Cells were harvested for flow cytometry analysis and apoptosis assay on day 4. **(B)** Flow cytometry analysis of the percentage of IL9R expressing A549 cells unstimulated, or stimulated with IFNɣ, IL21 or TNFα, respectively. A representative dot plot is shown for each group. **(C)** Flow cytometry analysis of the percentage of IFNɣR (CD119) expression A549 cells unstimulated or stimulated with IFNɣ, IL9, IL21 or TNFα, respectively. Both IL9 pretreated and untreated A549 cells were analyzed. A representative dot plot is shown for each group. **(D)** Experimental design of A549 cell line culture for 72 hours. A549 cells were untreated, or treated with IFNɣ (50 ng/ml) or IL9 (50 ng/ml) for 72 hours. In addition, one group was first pretreated with IFNɣ (50 ng/ml) for 24 hours, then stimulated with IL9 (50 ng/ml) for 48 hours. After 72 hours, all cells were harvested for flow cytometry analysis for cell apoptosis. Flow cytometry analysis of the percentage of A549 early apoptosis (Annexin V+PI- cells) in unstimulated cell culture condition or in condition of IFNɣ, IL9, IFNɣ+IL9, respectively. A representative dot plot is shown for each group. All data were statistically analyzed with ordinary one-way ANOVA or two-way ANOVA, and showed with mean ± SEM. n=3 per group. *P < 0.05; **P < 0.01; ***P < 0.001; ****P < 0.0001.

Finally, we performed FACS analysis in the human adenocarcinoma lung cancer cell line A549 to understand if IL-9R is expressed in these tumor cells and detected IL9R expression **(** **Figures 4A, B** **)** in A549 cells. The gating strategy for FACS staining is shown in **Supplementary Figure 3A**. In summary, subsets of tumor infiltrating lymphocytes in the lung as well as lung adenocarcinoma cells express IL9R and may thus be targets for immunomodulatory and cancer promoting effects of IL-9.

### IFN-ɣ Inhibited IL-9R on A549 but IL-9 Did Not Inhibit IFN-ɣ Induced Lung Tumor Apoptosis

We next asked if IFN-γ would regulate IL-9 signaling. Here we found that, IFN-ɣ, but not IL-21 and TNF-α treatment significantly downregulated IL-9R expression on A549 cells (**Figure 4B**). Moreover, independently from IL-9, we found that, TNF-alpha induced IFN-ɣ receptor (CD119) levels on A549 cells (**Figure 4C**), indicating an opposite role of IFN-ɣ and TNF-alpha as compared to IL-9 on lung tumor cells.

It has been reported that IL-9 induced A549 proliferation (36). Therefore, we investigated if IL-9 would influence the tumor cell survival and death. Here we found that, as opposed to the anti-tumoral cytokine IFN-ɣ, IL-9 suppressed lung tumor cell apoptosis **(** **Figure 4D** **)**. Furthermore, pre-treatment of A549 cells with IFN-ɣ reversed the anti-apoptotic effect of IL-9 **(** **Figure 4D** **).** Taken together these data indicate a pro-tumoral role of IL-9 in lung cancer.

### IFN-ɣ Induced and IL-9 Inhibited Fas Receptors (FAS-R) on Lung Cancer Cells

**Figure 5:**
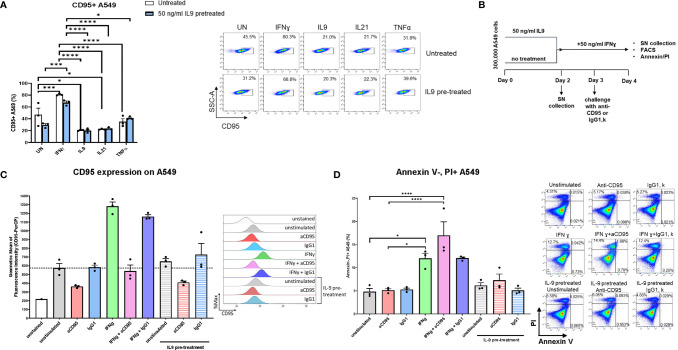
Analysis of IL9 effects on tumor cell survival *in vitro* on A549 cell line. **(A)** Flow cytometry analysis of the percentage of CD95 (Fas) expressing A549 cells unstimulated or stimulated with IFNɣ, IL9, IL21 or TNFα, respectively. Both IL9 pretreated and untreated A549 cells were analyzed**. (B)** Experimental design of A549 cell line culture for 4 days. **(C)** Flow cytometry analysis of the geometric mean of CD95 (Fas) expressing A549 cells unstimulated or stimulated with aCD95, IgG1, IFNγ and a combination of aCD95 or IgG1 with IFNγ, respectively. Furthermore, aCD95 and IgG1 stimulated cells were analyzed after IL-9 pre-treatment. **(D)** Flow cytometry analysis of the percentage of A549 necrotic cells (Annexin V-PI+ cells) in unstimulated cell culture condition or in condition of aCD95, IgG1, IFNγ and a combination of aCD95 or IgG1 with IFNγ, respectively. Furthermore, aCD95 and IgG1 stimulated cells were analyzed after IL-9 pre-treatment. A representative histogram (c) or dot plot (d) is shown for each group. The data were statistically analyzed with two-way ANOVA, and showed with mean ± SEM. n=3 per group. *P < 0.05; **P < 0.01; ***P < 0.001; ****P < 0.0001.

In search of the molecular mechanism of IFN-ɣ’s apoptotic effect on lung tumor cells, we discovered FAS-R (CD95) induction on A549 cells mediated by IFN-ɣ and an inhibition of FAS-R expression by IL-9 treatment (**Figure 5A**).

We next treated A549 cells either with IFN-ɣ or pretreated them with IL-9 and use agonistic antibodies to activate FAS-R (CD95). Here we could confirm that IFN-ɣ induced specifically FAS-R (**Figures 5B, C** **)**. Moreover, treatment with agonistic anti-CD95 antibodies resulted in induction of IFN-ɣ mediated A549 cell death (**Figure 5D**).

By contrast, agonistic FAS-R treatment did not affect the effect of IL-9 on apoptosis demonstrating that IFN-ɣ-FAS mediated apoptosis is not influenced by IL-9. To prove that IFN-ɣ mediated A549 apoptosis, but not IL-9 induced tumor apoptosis *via* FAS-R.

### IL-9R Is Induced in CD4+CD25+Foxp3+ Treg Cells During T Regulatory Cell Development

To further explore the effects of IL-9 on Treg cells, we analyzed IL-9 production and IL-9R expression by T regulatory cells differentiated *in vitro* in naïve mice. In addition, we added IL-9 and anti-IL-9 antibodies to see their effects on Tregs. As comparison we also set up Th9 skewing conditions. It was found that IL-9 is produced by Treg skewed lymphocytes although at lower levels as compared to Th9 cells **(** **Figure S4A, B** **)**. Moreover, Treg skewing conditions did not result in increased Th9 cells **(** **Figure S4C** **)**. In addition, Th9 skewing conditions induced Foxp3+ Treg cells **(** **Figure S4D** **)** although at lower levels as compared to Treg skewing conditions. IL-9 did not further induce Treg development upon IL-2 and TGF-beta stimulation **(** **Figure S4D** **)**. Finally, when naive T cells were differentiated into Treg cells, IL-9R expression mRNA was upregulated at day 5 as compared to day 3 **(** **Figure S4E** **)**. These findings suggest that Treg cells produce lower levels of IL-9 as compared to Th9 cells but may have increased capability to respond to IL-9 signaling as they differentiate. We then analyzed levels of IFN-ɣ, an anti-tumor Th1 cytokine released by different immunocompetent cells such as CD8+ T cells that play a key role in anti-tumor mediated immune responses (27, 37, 38). To this aim, we first skewed naïve spleen CD4+CD62L+ T cells under Th1 skewing conditions. As IL-9 production of naive CD4+ T cells is inhibited by IFN-ɣ (9, 39), we induced Th1 development in naïve spleen T cells by incubating naïve cells with IL-12, a cytokine produced by dendritic cells and macrophages that induces IFN-ɣ production in naive T cells (40), and anti-IL-4 antibodies. In these studies, we found that Th1 skewing conditions inhibited IL-9 while inducing IFN-ɣ levels in the supernatants **(** **Figure S4F** **).**

### In a Syngenic Model of Lung Cancer With LL/2 Cells IL-9 Deficiency Resulted in Decreased Tumor Load Associated With Increased IL-21 Levels

**Figure 6:**
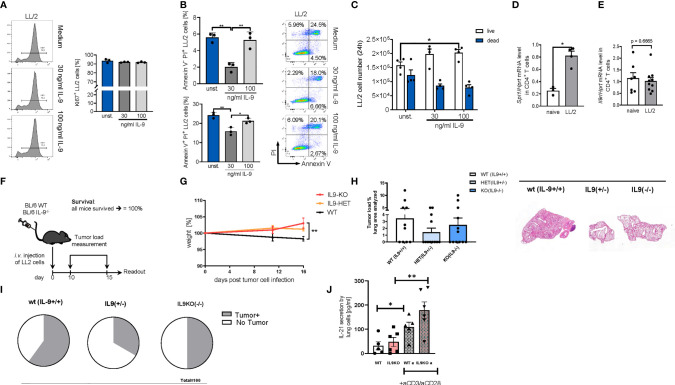
Targeted deletion of IL-9 resulted in lung tumor rejection in BL6 syngenic mice. **(A)** *In vitro* studies of murine LL/2-luc-M38 (LL/2) lung carcinoma cells treated with/out recombinant IL-9 for 24h. Flow cytometry analysis of the percentage of Ki67 expressing LL/2 cells after stimulation with or without IL-9 for 24h and representative histograms (left) (n unst. = 3; n30 ng/ml=3; n100 ng/ml=3). **(B)** Annexin V/PI flow cytometry analysis of LL/2 cells after stimulation with or without IL-9 for 24h and representative dot plots (n unst. = 3; n30 ng/ml=3; n100 ng/ml=3). **(C)** Cell count by using Neubauer chamber. Bar charts indicate mean values ± SEM using student´s two-tailed *t*-test (b–g) or ordinary one-way ANOVA (i–j) (*p<0.05, **p<0.01, ***p<0.001). **(D)** qPCR based analysis of *Spi1* encoding PU.1 (n_naive_=3; n_LL/2 =_ 4) and **(E)** *Il9r* (n_naive_=7; n_LL/2 =_ 10) in relation to Hprt mRNA level in CD4^+^ T cells isolated cells from the lung of naïve and lung tumor-bearing (LL/2) WT mice. **(F)** Experimental design for the induction of lung tumor development in BL/6 WT, BL/6-IL-9^+/-^ and BL/6 IL-9^-/-^ mice which were intravenously (i.v.) with LL/2-luc-M38 (LL/2) lung carcinoma cells on day 0. The experiment ended on day 16. **(G)** Monitored body weight during tumor development. **(H)** Quantification of the tumor-infiltrated area of the lung (left) and representative H&E staining of the lung of WT, IL-9^+/-^ and IL-9^-/-^ mice (right) (n_WT_=10; n _IL-9+/-=_10 n_IL-9-/-_=10). **(I)** Circles represent mice per group tumor free (circle sectors in white) and mice with lung bearing tumor (sectors in grey). **(J)** ELISA analysis of IL-21 levels (pg/ml) in supernatant obtained from cells isolated from the lung of wild-type and IL-9^-/-^ tumor-bearing mice. Lung cells were cultured for 4 days unstimulated or re-stimulated with aCD3/aCD28 antibodies (n_WT_=5, n_IL9-/-_=6).

In subsequent experiments, we performed studies in the syngenic LL/2 model and analyzed the direct anti-tumoral function of IL-9 on the tumor cell line LL/2 by using Ki67 and AnnexinV/PI as proliferation and apoptosis markers, respectively **(** **Figures 6A, B** **)**. By using Ki67 staining we found that, IL-9 stimulation did not induce tumor cell proliferation of LL/2 cells **(** **Figure 6A** **).** In addition, IL-9 significantly reduced late apoptosis in LL/2 cells at the lower concentration analyzed, as determined by Annexin/PI staining, suggesting that IL-9 may directly regulate apoptosis of LL/2 lung tumor cells in a dose dependent manner **(** **Figure 6B** **).** The overall survival of LL/2 was induced by higher concentrations of IL-9 at 100ng/ml **(** **Figure 6C** **).**

We next induced lung tumor in a syngenic model of disease in C57Bl/6 mice. As the transcription factor PU1 has been implicated in controlling IL-9 production by T cells (16), we then asked if levels of PU1 (*Spi1* mRNA) are upregulated in lung T cells of wild-type mice bearing tumors. In fact, *Spi1* mRNA levels were significantly induced in tumor infiltrating T cells in WT mice as compared to lung T cells from mice lacking tumors **(** **Figure 6D** **)**. In addition, we demonstrated that these lung T cells may respond to IL-9 stimulation because they express the IL-9R **(** **Figure 6E** **).** However, we could not find any difference in IL-9R expression between tumor infiltrating T cells and lung T cells from naïve mice.

In this syngenic model **(** **Figures 6F** **–J** **)**, we found that, targeted deletion of IL-9, resulted in induced body weight as compared to the wild type littermates bearing tumor **(** **Figures 6F, G** **).** Moreover, by assessing the tumor load, we found that both the IL-9 deficient and the IL-9 heterozygous mice had a decreased (but not statistically significant) tumor load as compared to wild type littermates **(** **Figure 6H** **)**. More importantly, 67% of the IL-9 heterozygous and 50% of the IL-9 knockout mice were tumor free as opposed to 40% in the wild type mice group, indicating an anti-tumoral effect of IL-9 deficient mice **(** **Figure 6I** **)**. By looking into the mechanism we found that consistent with the presence of Th9 cells in the tumor of NSCLC patients, targeted deletion of IL-9 resulted in significant upregulation of IL-21, a cytokine produced by Th9 cells that may regulate anti-tumor effects of Th9 cells in lung cancer **(** **Figure 6J** **)**.

### Targeted Deletion of IL-9 Reduced Tumor Load and Induced Tumor Free Lungs in a Second Syngenic Model of Lung Cancer

**Figure 7:**
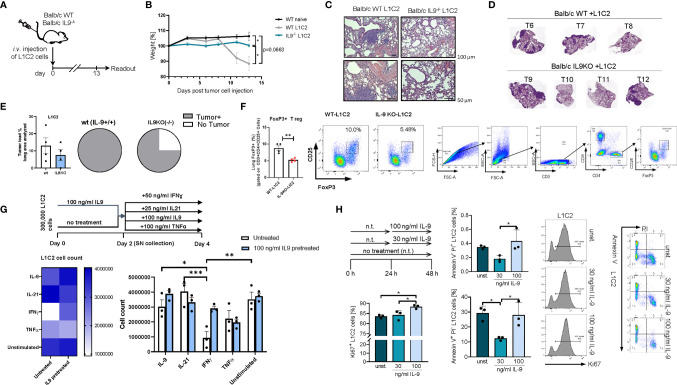
Targeted deletion of IL-9 resulted in lung tumor rejection Balb/c syngenic mice. **(A)** Experimental design for the induction of lung tumor development in Balb/c wild-type (WT) and Balb/c IL9^-/-^ mice. Tumor growth was induced *via* intravenous (i.v.) injection of L1C2 lung carcinoma cells. The experiment ended on day 14. **(B)** Body weight changes of Balb/c IL9^-/-^ mice and Balb/c control littermates (injected i.v. with/out L1C2 cells) 0 to 14 days after tumor induction (n_IL9-/-_=4; n_balb/c L1C2 =_ 3; n_balb/c naive_=2). **(C)** Representative H&E staining of the lung of WT and IL-9^-/-^ mice (top, scale bar=100 µm; bottom, scale bar=50 µm) and **(D, E)** additional mice were evaluated by a pathologist in a blind manner (n=4 per group). Circles represent mice per group tumor free (sectors in white) and mice with lung bearing tumor (sectors in grey). **(F)** FACS analysis of Foxp3+ CD25+ T regulatory cells in total lung cells of 4 wt and 4 IL9 ko mice after gating on CD3+CD4+ T cells. **(G)** Experimental design is similar to the A549 cell line culture whereby L1C2 were pre-cultured or not for 2 days of IL9 (100 ng/ml) and then further cultured for 2 days unstimulated or cultured with either IFNɣ, or IL9, IL21 or TNFα, respectively. The data were statistically analyzed with two-way ANOVA, and showed with mean ± SEM. n=3 per group. *, P<0.05; **, P<0.01; ***, P<0.001; ****, P<0.0001. **(H)** Flow cytometry analysis of the percentage of Ki67 expressing L1C2 cells after stimulation with or without IL-9 for 24h and representative histograms (n-unst. = 3; n30 ng/ml=3; n100 ng/ml=3). Annexin V/PI flow cytometry analysis of L1C2 cells after stimulation with or without IL-9 for 24h and representative dot plots (n-unst. = 3; n30 ng/ml=3; n100 ng/ml=3). Bar charts indicate mean values ± SEM using student´s two-tailed t-test (b-g) or ordinary one-way ANOVA (i-j) (*p < 0.05, **p < 0.01, ***p < 0.001).

We next asked if the presence of IL-9 affects tumor development in a second syngenic model of lung cancer after intravenous injection of L1C2 lung tumor cell line in a Balb/c genetic background **(** **Figure 7** **)**. Consistent with the other models of disease, we confirmed significant upregulation of body weight in the absence of IL-9 during tumor development as compared to wild type littermates **(** **Figures 7A, B** **).** We observed a reduction of the tumor load, by trend **(** **Figures 7C–E**). Similar to the LL/2 model, we observed in additional experiments that the number of mice with no tumor was induced in the absence of IL-9 **(** **Figure 7E** **)**. Moreover, the immunosuppressive FoxP3+CD4+CD25+ T regulatory cells were reduced in the absence of IL-9 **(** **Figure 7F** **)**, consistent with our finding that Treg cells express IL-9R. Finally, we directly analyzed the pro-tumoral function of IL-9 on the tumor cell line L1C2 by using cell counts **(** **Figure 7G** **)**, Ki67 **(** **Figure 7H** **)** and AnnexinV/PI **(** **Figure 7I** **)** as proliferation and apoptosis markers, respectively. By using cell counts, we found that IL-9 did not influence the tumor cell count, while IFN-ɣ and TNF-alpha decreased the overall tumor cell count **(** **Figure 7G** **)**. By Ki67 staining, we detected that IL-9 stimulation significantly induced L1C2 tumor cell proliferation in a dose dependent manner **(** **Figure 7H** **).** In addition, IL-9 significantly reduced early apoptosis in L1C2 cells at lower concentrations, as determined by Annexin/PI staining, suggesting that IL-9 may directly regulate both apoptosis and proliferation of L1C2 lung tumor cells **(** **Figure 7H** **)**.

### Anti-IL-9 Antibodies Inhibit Lung Tumor Growth in the Syngenic LL/2 Model *In Vivo*

**Figure 8:**
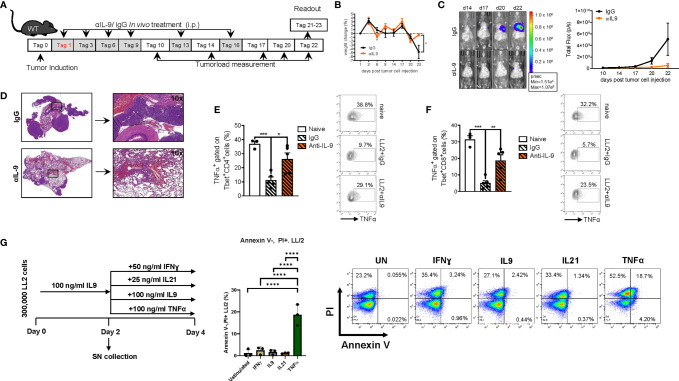
Anti-IL9 antibody treatment inhibits lung tumor development. **(A)** Experimental design: BL/6 WT mice were intravenously injected with LL/2 cells on day 0. Mice were then treated intraperitoneally (i.p.) with anti (α)-IL9 antibody or IgG2a isotype control on days 1, 3, 6, 9, 13 and 16 after tumor induction. Overall survival rate of αIL9 antibody or IgG2a isotype control treated mice. Survival rate on the right handside. **(B)** Monitoring of body weight changes of BL/6 WT mice injected i.v. with LL/2 cells). **(C)** Tumor load was analyzed *via* bioluminescence-based imaging system on the indicated days. The experiment ended on day 23. Representative *in vivo* images of lung tumor load analysis at days 14, 17, 20 and 22 in WT mice treated with αIL9 antibody or IgG2a isotype control (representative of 3 independent experiments) are shown. and lung tumor load (Total flux=photons/second) (Day10-20 n_IgG2a+LL/2 =_ 5, n_αIL-9+LL/2 =_ 5; day22 n_IgG2a+LL/2 =_ 5, n_αIL-9+LL/2 =_ 4) were quantified. **(D)** Representative H&E staining of WT mice treated with αIL9 antibody or IgG2a isotype control (scale bar=200 µm). **(E)** Flow cytometry analysis of TNFα positive cells (%) gated on Tbet and CD4 co-expressing cells (nnaive=3; nIgG2a+LL/2 = 5; nαIL-9+LL/2 = 5). **(F)** Flow cytometry analysis of TNFα positive cells (%) gated on Tbet and CD8 co-expressing cells (nnaive=3; nIgG2a+LL/2 = 5; nαIL-9+LL/2 = 5). **(G)** Experimental design is similar to the A549 cell line culture whereby LL/2 were pre-cultured for 2 days of IL9 (100 ng/ml) and then further cultured for 2 days unstimulated or cultured with either IFNɣ, or IL9, IL21 or TNFα, respectively. Annexin V(AnnV)/PI flow cytometry analysis of IL9 pre-treated LL/2 cells at the end of the cell culture, A representative dot plot is showed for each group. The data were statistically analyzed with one-way ANOVA, and showed with mean ± SEM. n=3 per group. *P < 0.05; **P < 0.01; ***P < 0.001; ****P < 0.0001.

The above findings are consistent with a model in which IL-9 derived by TIL cells directly favours tumor cell survival and suppresses anti-tumor immune responses by effects back on TILs. Therefore, we asked if targeting of IL-9 function might be therapeutically useful in lung cancer. To explore this concept under *in vivo* conditions, we next studied the effects of neutralizing anti-IL-9 antibodies in our experimental lung cancer model induced by LL/2 cells *in vivo.* To this aim, we blocked IL-9 repeatedly during lung tumor development in WT mice *via* intraperitoneal injection of anti-IL-9 antibodies **(** **Figure 8A** **)**. Consistent with the phenotype discovered for IL-9 deficient mice, intraperitoneal anti-IL-9 antibody treatment in WT mice resulted in increased body weight and an increased tumor rejection **(** **Figures 8B–D**) as compared to control IgG-treated WT animals. Moreover, we found that IgG control treatment in tumor model resulted in decreased number of CD4+ and CD8+ T-bet+TNF-α+ T cells which were induced by the anti-IL-9 antibody treatment **(** **Figures 8E, F** **)**. Finally, we pretreated LL/2 cells with IL-9 for 48 hours and then challenge LL/2 cells with different cytokines. Shown is flow cytometric analysis of the percentage of necrotic cells (Annexin V-PI+ cells) after IL9 (100 ng/ml) pretreatment of LL/2 cells following 48h unstimulated cell culture condition or in conditions with IFNɣ, IL9, IL21, TNFα treatment, respectively. Under these conditions only TNF-α showed a pro-apoptotic function on LL/2 tumor cells **(** **Figure 8G** **)**.

### Blockade of IL-9 at Later Time Points in Established Tumors Resulted in Tumor Rejection in the Two Syngenic LL/2 and L1C2 Mediated Models of Experimental NSCLC in Mice

**Figure 9:**
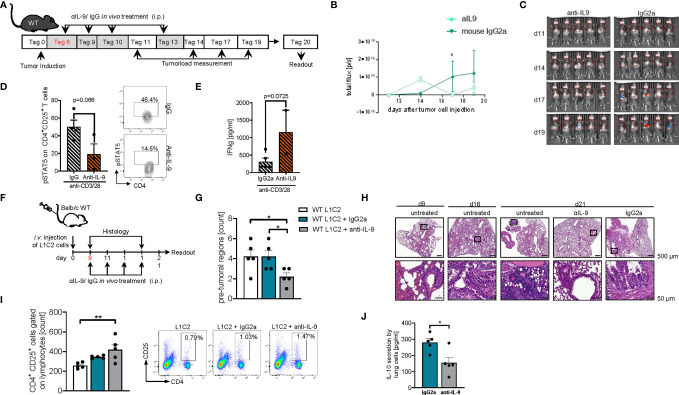
Later treatment with anti-IL9 antibodies during tumor development, protected from experimental induced tumor. **(A)** Experimental design: BL/6 WT mice were intravenously injected with LL/2 cells on day 0. Mice were then treated intraperitoneally (i.p.) with anti (α)-IL9 antibody or IgG2a isotype control on day 6, 9, 10 and 13 after tumor induction. The experiment ended on day 20. **(B, C)** Tumor load was analyzed *via* bioluminescence-based imaging system on the indicated days. Representative *in vivo* images of lung tumor load analysis at day 11, 14, 17 and 19 in WT mice treated with αIL9 antibody or IgG2a isotype control (c). (b)Quantification of lung tumor load (Total flux=photons/second) (Day10-20 n_αIL-9+LL/2 =_ 5; n_IgG2a+LL/2 =_ 5). **(D)** FACS analysis of lung pSTAT5+CD4+CD25+ lung cells after 24 hours culture with anti_CD3/CD28 antibodies (n-αIL-9+LL/2 = 5; n-IgG2a+LL/2 = 5). **(E)** ELISA analysis of IFNγ levels (pg/ml) in supernatants obtained from cells isolated from the lung of wild-type (WT) lung tumor-bearing mice treated with/out anti(α)-IL9 antibody or IgG2a isotype control and cultured for 24h at 37°C (nIgG2a= 5; nαIL-9 = 5). N values are given per group. Bar charts indicate mean values ± SEM using student´s two-tailed t-test *p<0.05, **p<0.01, ***p<0.001. **(F)** Experimental design: Balb/c WT mice were intravenously injected with L1C2 cells on day 0. Mice were then treated intraperitoneally (i.p.) with anti (α)-IL9 antibody or IgG2a isotype control on day 9, 11, 14 and 16 after tumor induction. Tumor load was analyzed *via* immune histology on the indicated days. The experiment ended on day 21. **(G, H)** Representative H&E staining of WT mice untreated or treated with αIL9 antibody or IgG2a isotype control on day 9, 16 and 21 (top, scale bar=500 µm; bottom, scale bar=50 µm) and quantification of the number of pre-tumoral lesions in the in the lung on day 21 (_nIgG2a_= 5; n_αIL-9 =_ 5). **(I)** Flow cytometry analysis of percentage of CD4+CD25+ cells gated on lymphocytes in non-treated or with αIL9 antibody or IgG2a isotype control treated mice. **(J)** ELISA analysis of IL-10 levels (pg/ml) in supernatant obtained from cells isolated from the lung of wild-type (WT) lung tumor-bearing mice treated with/out anti(α)-IL9 antibody cultured for 24h at 37°C (n_untr. =_ 5; _nIgG2a_= 5; n_αIL-9 =_ 5). N values are given per group. Bar charts indicate mean values ± SEM using student´s two-tailed *t*-test *p < 0.05, **p < 0.01, ***p < 0.001.

In subsequent studies on the effects of anti-IL-9 antibodies in experimental NSCLC, we explored the effects of these antibodies at later time points of tumor induction in the two models of disease in syngenic mice. In the LL/2 model we started the treatment at day 6 after tumor cell injection **(** **Figure 9A** **).** Here we observed that, blocking IL-9 at later time points during tumor development resulted in a significant tumor reduction as compared to IgG treatment **(** **Figures 9B, C** **).** pSTAT5 is activated downstream of IL-9 and thus we looked at pSTAT5 in lung CD4+CD25+ T cells and found that this cell population was down-regulated by anti-IL-9 antibody treatment **(** **Figure 9D** **).** By trend, we also found induction of IFN-ɣ after anti IL-9 treatment (**Figure 9E** **).**

We next analyzed a second syngenic model of disease using the cell line L1C2 in a BALB/c genetic background and started treatment at a later time point at day 9 when the tumor was already present in the lung **(** **Figure 9F** **)**. In this model we found a reduction of the number of pre-tumoral lesions in the anti-IL-9 antibody treated mice **(** **Figure 9G** **).** Additionally, we studied the number of activated CD4+CD25 (IL2Ralpha)+ T cells and found them to be induced in the lung of anti-IL-9 antibody treated mice **(** **Figure 9I**). Moreover, the immunosuppressive cytokine IL-10 was downregulated upon anti-IL-9 treatment **(** **Figure 9J** **),** suggesting that IL-9 controls the balance between inflammatory and immunosuppressive cytokines in NSCLC.

## Discussion

In this study, we investigated the functional role of IL-9 in NSCLC. We observed an induction of IL-9 production by tumor infiltrating lymphocytes (TIL) and a subset of FoxP3+ regulatory T cells in the tumoral region of NSCLC patients and identified tumor infiltrating T cells, FoxP3+ Treg cells and tumor cells as IL-9R expressing target cells for IL-9 signaling. The functional relevance of IL-9 signaling in lung cancer was underlined by studies in several independent murine NSCLC models of lung adenocarcinoma where IL-9 deficiency resulted in suppression of tumor growth. Finally, antibodies to IL-9 inhibited tumor growth *in vivo* in two syngenic models of disease. These findings suggest that targeting of IL-9 function may be useful for immunotherapy of NSCLC.

In an initial series of studies on the role of IL-9 in NSCLC, we analyzed samples from a large cohort of NSCLC patients. In the lung of patients with NSCLC, we found IL-9+ cells induced in the tumoral region of the lung as compared to the control region **(** **Figure 1B** **).** While no induction of epithelial cells expressing IL-9 was noted in NSCLC patients, we identified tumor infiltrating T cells in NSCLC as important IL-9 producers **(** **Figures 1G, H** **)**. Thus, we determined IL-9 production in different T cell subsets **(** **Figure 2** **).** Upon antigen binding, naïve CD4^+^ T cells can differentiate into distinct subtypes, such as Th1, Th2, Th9, and Th17 cells but also regulatory T cells (Tregs) (41–43). The development of each T cell subtype can be induced by a specific cytokine milieu and is characterized by the expression of lineage specific hallmark transcription factors, such as Tbet, Gata3, Rorγt or STAT5 and Foxp3 (44–48). Here, we noted that IL-9 is increasingly produced by TIL **(** **Figures 1G, H** **)** and a subset of Foxp3 expressing Treg cells **(** **Figures 2A–D**) in lung tissues of NSCLC patients. Although IL-9 producing Treg cells have been described in inflammatory conditions such as nephritis (49), they have not been observed in cancer tissue so far. However, we found that such Foxp3+ T cells are located in lung cancer tissue of NSCLC patients. As FoxP3+ Treg cells are known to suppress anti-tumor immunity in NSCLC (50), these findings suggested that IL-9 derived from Treg cells and TIL might control immune responses and tumor growth. As TIL in NSCLC patients produced elevated amounts of the Th9 cytokine IL-21 (7, 22) and expressed the Th9 associated transcription factor PU.1 (15, 28), our findings suggested that Th9 cells as well as Tregs are an important source of IL-9. Future studies on the relative contribution and functional relevance of these IL-9 producing T cell subsets (for instance, by using conditional IL-9 deficient animals) are warranted.

Further studies suggested that IL-9 derived from tumor infiltrating T cells (TILs) as well as the tumor cells themselves might influence the T regulatory cells infiltrating the tumor. In fact, TILs were found to release induced IL-9 upon antiCD3/CD28 antibody treatment **(** **Figure 1H** **),** and T regulatory cells had increased levels of IL-9R *ex vivo* **(** **Figure S4** **)**. Mechanistic studies in a murine model of lung adenocarcinoma highlighted the functional relevance of IL-9 signaling for cancer growth *in vivo*. Specifically, we found that the absence of IL-9 inhibits lung carcinoma growth and drives tumor rejection in two independent murine models of NSCLC.

These findings led to further mechanistic studies showing decreased numbers of T regulatory cells expressing Foxp3 in the absence of IL-9 in one model of disease **(** **Figure 7F** **)** confirming the predominant effects of IL-9 on the presence and function of immunosuppressive T cells that facilitate cancer growth. Altogether, these data support a pro-tumoral function of IL-9 in TILs that may be considered for the design of new immunotherapies against NSCLC. In fact, we demonstrated here that anti-IL-9 antibodies given intraperitoneally during tumor development resulted in increased survival rates and enhanced tumor rejection **(** **Figures 8**, **9** **)**. Remarkably, anti-IL9 treatment was even effective in mice with already established lesions in both models suggesting a key function role of IL-9 in controlling tumor growth *in vivo*. In the syngenic L1C2 model, IL-9 blockade resulted in a downregulation of IL-10 **(** **Figure 9J** **)**. As IL-10 and TGF-beta expressing T cells are known to play a crucial role for inducing Foxp3+ T regulatory cells and controlling immune responses in NSCLC (31, 35), these results suggest that IL-9 plays an important role in regulating immune responses in lung cancer *in vivo*.

Further studies on the effects of IL-9 blockade in experimental lung cancer identified direct effects of IL-9 on tumor cells. Specifically, human lung cancer cells expressed the IL-9R **(** **Figures 4A, B** **)**. Furthermore, IL-9 treatment prevented the induction of tumor cell death in lung adenocarcinoma cells indicating a pro-tumoral role of IL-9 signaling. Experimental studies in NSCLC cell lines identified effects of IL-9 on cell proliferation or apoptosis suggesting that this cytokine drives tumor growth in NSCLC both in human **(** **Figure 5A** **)** and in murine tumor cells **(** **Figure 7H** **).** Consistently, a previous study showed that IL-9 promoted the proliferation of A549 lung cancer cells (36). Moreover, IL-9 facilitated intercellular adhesion of these cancer cells to pleural mesothelial cell monolayers suggesting that this cytokine may be involved in malignant pleural effusion and pleural metastasis (36).

Reports on the role of IL-9 and Th9 cells in tumor immunity have yielded partially controversial results (51–54). While an important anti-tumoral role of IL-9 was found in melanoma, we observed here a key pro-tumoral role of IL-9 in NSCLC. One possible explanation for these differences relates to IL-9 target cells in different tumor types. In fact, we noted here that IL-9 controls tumor cell growth in NSCLC *via* direct effects on IL-9R expressing cancer cells. In contrast, IL-9 effects on anti-cancer immunity *via* the tumor micromilieu rather than direct effects on tumor cells were noted in melanoma. Additional differences could be due to discrepancies in the tumor microenvironment between melanoma and NSCLC or the analysis of IL-9- versus Th9-dependent effects, as many studies used Th9 cell transfer-models rather than IL-9 single cytokine targeting to determine the functional relevance of IL-9. However, Th9 cells produce IL-21 in addition to IL-9, a cytokine with anti-tumor function. Recently, it was report that T follicular helper cells producing IL-21 play crucial roles in lung adenocarcinoma, because these cells were able to induce the effect function of tumor-infiltrating CD8 *via* IL-21/IL-21R signaling (55). Moreover, it has been reported that the transcription factor IRF1 enhanced the effector function of Th9 cells and dictated their anticancer properties. Consistent with this, IRF1 inhibits IRF4 effects on the IL-9 promoter (23). These observations raise the possibility that selective targeting of IL-9 may yield different results as compared to Th9 cell targeting, as the anti-tumor effect of the Th9 cytokine IL-21 is not targeted by anti-IL-9 antibodies. This possibility was also confirmed by the upregulation of IL-21 in a second syngenic model of disease in IL-9 ko mice bearing tumor **(** **Figure 6J** **).** Thus, the role of IL-9 must be revised and analyzed independent from Th9 cells. Taken together, our findings indicate effects of IL-9 on growth of lung cancer cells. This concept is in agreement with the finding that IL-9 promotes the development of many hematological human tumors, including Hodgkin’s lymphoma and B cell lymphoma (56) suggesting the presence of pro-tumoral effects of IL-9 in several tumor entities.

In summary, the present study has uncovered an important pro-tumoral role of IL-9 in NSCLC *via* direct effects on tumor cells and modulation of cytokine production by tumor infiltrating T cells. This observation in NSCLC is not consistent to the previously reported anti-tumoral role of IL-9 in melanoma where decreased IL-9 levels were associated with tumor growth (54, 57). Although the precise reasons for these differences remain currently unclear, they may be related to the specific microenvironment provided by lung TILs in NSCLC or to the question whether the targeted tumor cell type expresses the IL-9R. The expression of IL-9R on lung adenocarcinoma cells may permit direct effects of IL-9 on tumor cell proliferation or apoptosis. Consistent with these data, we noted that IL-9 treatment augments tumor cell proliferation or prevented cell death of human NSCLC cell lines in cell culture. Moreover, our findings demonstrate that anti-IL-9 antibodies markedly suppress tumor growth *in vivo* and identify IL-9R+ TILs and lung adenocarcinoma cells as targets for IL-9 signaling. Our findings provide new insights into the regulatory role of IL-9 for lung cancer growth and suggest new avenues for therapy of NSCLC by targeting of IL-9 function.

## Data Availability Statement

The raw data supporting the conclusions of this article will be made available by the authors, without undue reservation.

## Ethics Statement

The studies involving human participants were reviewed and approved by the ethics review board of the University of Erlangen (Re-No: 56_12B; DRKS-ID: DRKS00005376). The patients/participants provided their written informed consent to participate in this study. The animal study was reviewed and approved by Ethics committees of the Regierung Unterfranken (Az 55.2-2532-2-1286-20).

## Author Contributions

SF designed the experiments, supervised the research, performed western blot and analysis for Human IL-9 in **Figure 1E**, murine 6I and **7E**, helped with set up human IHC, WB and their analysis, helped with data analysis and wrote this manuscript. LH analysed most of the human lung data, contributed to the murine anti-IL-9 therapy, and to the writing of the manuscript. ZY reanalyzed all the human data, generated the A549, LL/2 and L1C2 figures, except for **Figures 4D**, generated by SM and **Figures 6A–C** and **7H** which were generated by JK and helped with the manuscript writing and generation of the figures. PT and KH did all the FACS and ELISA analysis in human PBMCs samples, contributed to western blot and IL9 -/- mice analysis as well as to revisioning the manuscript. PT contributed to the anti-FAS mediated A549 apoptosis and relative FACS analysis and set up tumor load analysis in murine model. C-IG is the pathologist that digitalized all the histological sections (murine and human) and contributed to the tumor load analysis. KK, AG, JF, and SM helped with the murine anti IL-9 therapy (LL/2) and the human NSCLC lung FACS and IHC data. MTC injected the murine tumor cells i.v. JK helped with the anti IL9 therapy on Balb/c and did murine cell line culture and assisted to the generations of figures and material and methods. RK generated the Figure on murine TH9 and T reg differentiation. MK and MFN contributed to the writing of the manuscript. DIT and HS are the thoracic surgeons that performed human lung tumor surgery and RJR is the pathologist that generated the lung tissues arrays from NSCLC patients, as well as lung tissues from controls. In addition, he made the histopathologic definition of tumor free tissue (Control region) and Tumor cell containing tissue (Tumoral tissue) in the lung tissue arrays. All authors performed experiments, discussed the results or provided help with regard to the writing of the manuscript. All authors contributed to the article and approved the submitted version.

## Funding

This work was supported by the DFG-FI817-5/3 grants and by the Molecular Pneumology department in Erlangen. MC was supported by the DFG CH1428/2-1 and MK was supported by NIH grant AI057459. PT is supported by the Sander Stiftung 2020.016.1 awarded to SF, and ZY is supported by the SFB CRC1181 Project B08 N awarded to SF.

## Conflict of Interest

The authors declare that the research was conducted in the absence of any commercial or financial relationships that could be construed as a potential conflict of interest.

## Publisher’s Note

All claims expressed in this article are solely those of the authors and do not necessarily represent those of their affiliated organizations, or those of the publisher, the editors and the reviewers. Any product that may be evaluated in this article, or claim that may be made by its manufacturer, is not guaranteed or endorsed by the publisher.
