# Supplementary material for: IL-9 Producing Tumor-Infiltrating Lymphocytes and Treg Subsets Drive Immune Escape of Tumor Cells in Non-Small Cell Lung Cancer
Source: Front Immunol. 2022 Apr 20;13:859738. doi: 10.3389/fimmu.2022.859738 (PMC9065342; doi:10.3389/fimmu.2022.859738)
Supplement: Supplementary file 1 [file DataSheet_1.docx]

Supplementary Material

IL-9 producing tumor-infiltrating lymphocytes (TIL) and Treg subsets drive immune escape of tumor cells in non-small cell lung cancer

**Lisanne Heim^1*^, Zuqin Yang^1*^, Patrick Tausche^1^, Katja Hohenberger^1^ _,_ Mircea T. Chiriac^2^, Julia Koelle^1^, Carol-Immanuel Geppert^4,§^, Katerina Kachler^1^, Sarah Mitsch^1^ _,_ Anna Graser^1^, Juliane Friedrich^1^, Rakshin Kharwadkar^3^, Ralf J. Rieker^4,§^, Denis I. Trufa^5,§^, Horia Sirbu^5,§^, Markus F. Neurath^2,&,§^, Mark H. Kaplan^3^, and Susetta Finotto^1,§**^**

^1^ Department of Molecular Pneumology, Friedrich-Alexander-Universität Erlangen-Nürnberg (FAU), Erlangen, Germany.

^2^ Department of Internal Medicine 1, Friedrich-Alexander-Universität Erlangen-Nürnberg (FAU), Erlangen, Germany

^3^ Department of Microbiology and Immunology, Indiana University School of Medicine, Indianapolis, USA .

^4^ Institute of Pathology, Friedrich-Alexander-Universität Erlangen-Nürnberg (FAU), Erlangen, Germany.

^5^ Department of Thoracic Surgery, Friedrich-Alexander-Universität Erlangen-Nürnberg (FAU), Erlangen, Germany.

*These authors contributed equally to this work and thus share the first authorship.

^§^Comprehensive Cancer Center Erlangen-EMN (CCC ER-EMN), Erlangen, Germany

^&^ Deutsches Zentrum Immuntherapie DZI

**** Correspondence:**Prof. Dr. Dr. Susetta Finotto

Universitätsklinikum Erlangen

Abt. Molekulare Pneumologie

Hartmannstraße 14

91052 Erlangen

Phone: +49-9131-85-35883

[Mail: susetta.finotto@uk-erlangen.de](mailto:susetta.finotto@uk-erlangen.de)

[http://www.molekulare-pneumologie.uk-erlangen.de](http://www.molekulare-pneumologie.uk-erlangen.de/)

# Supplementary Figures and Tables

## Supplementary Table S1: Clinical patient data.

| Patient | Histological Classification | Tumour Ø (cm) | T | N | M | TNM Stadium | Gender | Age | Average Smoking (P/Y) |
| --- | --- | --- | --- | --- | --- | --- | --- | --- | --- |
| 1-MP | SCC | 1.3 | 1b | 0 | 0 | IA | Male | 80 | 40 |
| 2-MP | SCC | 5.1 | 1a | 0 | 0 | IA | Male | 57 | 40 |
| 3-MP | ADC | 5 | 2a | 0 | 0 | IB | Male | 79 | 60 |
| 4-MP | SCC | 2 | 1b | 1 | 0 | IIA | Female | 53 | 25 |
| 5-MP | SCC | 10.5 | 3 | 1 | 0 | IIB | Female | 67 | 50 |
| 6-MP | MTS ADC | 4.0 | # | # | # | # | Female | 79 | 0 |
| 7-MP | MTS SCC | 3.5 | # | # | # | # | Male | 45 | 1 |
| 8-MP | SCC | 5.5 | 3 | 0 | 0 | IIB | Male | 66 | 30 |
| 9-MP | ADC | 2.7 | 1b | 2 | 0 | IIIA | Female | 84 | 0 |
| 13-MP | SCC | 3 | 1b | 0 | 0 | IA | Male | 69 | 50 |
| 14-MP | SCC | 1.9 | 1a | 0 | 0 | IA | Female | 58 | 30 |
| 15-MP | ADC | 2.5 | 1b | 0 | 0 | IA | Male | 63 | 100 |
| 16-MP | ADC | 4.6 | 3 | 0 | 0 | IIB | Female | 70 | 15 |
| 17-MP | ADC | 2.6 | 2 | 0 | 0 | IB | Male | 74 | 70 |
| 18-MP | MTS ADC | 6.4 | # | # | # | # | Female | 52 | 2 |
| 19-MP | ADC | 6.5 | 2b | 0 | 0 | IIA | Female | 55 | 30 |
| 20-MP | ADC | 2.8 | 1b | 0 | 0 | IA | Male | 65 | 60 |
| 21-MP | SCC | 2.5 | 1b | 0 | 0 | IA | Male | 41 | 10 |
| 22-MP | ADC | 7 | 2b | 1 | 0 | IIB | Male | 68 | 82 |
| 23-MP | ADC | 4.5 | 2a | 0 | 0 | IB | Male | 73 | 75 |
| 26-MP | ADC | 1.3 | 1a | 0 | 1 | IV | Female | 52 | 50 |
| 27-MP | ADC | 1.4 | 1a | 0 | 0 | IA | Female | 70 | 50 |
| 28-MP | ADC | 1.2 | 1a | 0 | 0 | IA | Male | 76 | 60 |
| 29-MP | SCC | 3.7 | 1b | 0 | 0 | IIB | Male | 74 | 100 |
| 30-MP | SCC | 1.8 | 1a | 0 | 0 | IA | Female | 70 | 30 |
| 32-MP | ADC | 4.4 | 2a | 2 | 0 | IIIA | Female | 60 | 30 |
| 34-MP | ADC | 1.8 | 1 | 0 | 0 | I A | Female | 51 | 45 |
| 35-MP | ADC | 3 | 1b | 0 | 0 | IA | Female | 72 | 0 |
| 36-MP | SCC | 3.5 | 2a | 1 | 0 | IB | Male | 74 | 40 |
| 37-MP | SCC | 3.3 | 2a | 1 | 0 | IIA | Male | 60 | 45 |
| 39-MP | ADC | 6 | 2b | 0 | 0 | IIA | Male | 65 | 42 |
| 40-MP | ADC | 1.8 | 1a | 1 | 0 | IIA | Male | 82 | 100 |
| 44-MP | ADC | 1.5 | 1a | 0 | 0 | IA | Male | 53 | 70 |
| 45-MP | ADC | 2.3 | 1b | 0 | 0 | IA | Male | 78 | 0 |
| 51-MP | ADC | 2.4 | 1b | 2 | 1 | IV | Male | 62 | 90 |
| 52-MP | ADC | # | # | # | # | # | # | # | # |
| 53-MP | ADC | 2.25 | 1a | 0 | 0 | IA | Male | 62 | 10 |
| 55-MP | ADC | 1.8 | 1a | 2 | 0 | IIIA | Female | 64 | 40 |
| 56-MP | ADC | 4 | 2a | 0 | 0 | IB | Female | 67 | 0 |
| 57-MP | ADC | 3.8 | 2a | 0 | 0 | IB | Female | 35 | 10 |
| 58-MP | ADC | 6.5 | 3 | 0 | 0 | IIB | Female | 69 | 0 |
| 59-MP | ADC | 0.9 | 4 | 0 | 0 | IIIA | Male | 70 | # |
| 60-MP | SCC | 2.5 | 1b | 1 | 0 | IIA | Male | 71 | # |
| 62-MP | ADC | 3.5 | 1b | 0 | 0 | IA | Female | 80 | # |
| 64-MP | ADC | 3.5 | 1b | 0 | 0 | IA | Male | 55 | 35 |
| 65-MP | SCC | 2.8 | 1b | 0 | 0 | IA | Female | 76 | # |
| 71-MP | ADC | 3.7 | 2a | 0 | 0 | IB | Male | 67 | 75 |
| 73-MP | ADC | 4.8 | 2a | 0 | 0 | IB | Female | 67 | 22 |
| 74-MP | ADC | 3.2 | 2a | 0 | 0 | IB | Female | 58 | # |
| 75-MP | SCC | 4.8 | 2a | 1 | 0 | IIA | Male | 54 | 35 |
| 76-MP | SCC | 3.1 | 2a | 0 | 0 | IB | Male | 65 | 40 |
| 77-MP | ADC | 0.9 | 1a | 0 | 0 | IA | Female | 64 | 45 |
| 78-MP | ADC | 2.1 | 1b | 2 | 0 | IIIA | Female | 80 | 0 |
| 79-MP | SCC | 10 | 3 | 0 | 0 | IIB | Female | 67 | 50 |
| 80-MP | ADC | 5.4 | 4 | 0 | 0 | IIIA | Male | 62 | 28 |
| 81-MP | ADC | 1.6 | 1a | 0 | 0 | IA | Male | 61 | 46 |
| 82-MP | SCC | 8.5 | 3 | 0 | 0 | IIB | Male | 82 | 24 |
| 83-MP | ADC | 5.5 | 3 | 0 | 1a | IVA | Female | 60 | 0 |
| 84-MP | SCC | 7.2 | 2b | 0 | 0 | IIA | Female | 55 | 45 |
| 89-MP | ADC | 3.2 | 2 | 2 | 0 | IIIA | Female | 61 | 0 |
| 91-MP | ADC | 3.6 | 2a | 2 | 0 | IIIA | Male | 67 | 0 |
| 93-MP | ADC | 2.8 | 2a | 1 | 0 | IIIA | Male | 66 | 32.5 |
| 94-MP | ADC | 2.2 | 1c | 0 | 0 | IA3 | Female | 77 | 35 |

**Abbreviations:** ADC=adenocarcinoma; SCC=squamous cell carcinoma of the lung; MTS=metastasis; P/Y=Package/Year

**T-primary tumour:** 0: No evidence of primary tumour; 1a: Tumour 2 cm or less in greatest dimension; 1b: Tumour more than 2 cm but not more than 3 cm in greatest dimension; 2a: Tumour more than 3 cm but not more than 5 cm in greatest dimension; 2b: Tumour more than 5 cm but not more than 7 cm in greatest dimension; 3: Tumour more than 7 cm; **N-regional lymph nodes:** 0: No regional lymph node metastasis; 1: Metastasis in ipsilateral peribronchial and/ or ipsilateral hilar lymph nodes and intrapulmonary nodes, including involvement by direct extension; 2: Metastasis in ipsilateral mediastinal and/or subcarinal lymph node(s); **M-distant metastasis:** 0: No distant metastasis; 1: Distant metastasis.

# No information available

## Supplementary Table S2. Clinical data of the cohort of control subjects analysed in this study.

| **Sample**  **ID** | **Histological Classification** | **Tumour Ø (cm)** | **Grading** | **T** | **N** | **M** | **TNM Stadium** | **Average Smoking (P/Y)** |
| --- | --- | --- | --- | --- | --- | --- | --- | --- |
| CN-1 | Pneumothorax | 0 | / | / | / | / | / | # |
| CN-2 | Chondroid Hamartoma | 0 | / | / | / | / | / | # |
| CN-3 | Pneumothorax | 0 | / | / | / | / | / | # |
| CN-4 | Chondromatous Hamartoma | 0 | / | / | / | / | / | # |
| CN-5 | Pneumothorax | 0 | / | / | / | / | / | # |
| CN-6 | Rheumatoid Granuloma | 0 | / | / | / | / | / | # |
| CN-7 | Sarcoidosis | 0 | / | / | / | / | / | # |
| CN-8 | Pneumothorax | 0 | / | / | / | / | / | # |
| CN-9 | Chondroid Hamartoma | 0 | / | / | / | / | / | # |
| CN-10 | Pneumothorax | 0 | / | / | / | / | / | # |

Abbreviations: # No information available; / non-existing.

## Supplementary Table S3: List of subject analyzed in PBMCs studies.

| **Nr.** | **Patient** | **ADC/SCC** | **Grading** | **Age** | **Gender** |
| --- | --- | --- | --- | --- | --- |
| HC 1 | iK3 | Healthy Controls | | 25 | Male |
| HC 2 | iK1 |  |  | 53 | Female |
| HC 3 | iK4 |  |  | 30 | Male |
| HC 4 | iK9 |  |  | 25 | Female |
| HC 5 | C-508 |  |  | 30 | Female |
| NSCLC 1 | MP-149 | ADC | G2 | 69 | Male |
| NSCLC 2 | MP-150 | ADC | G2 | 64 | Male |
| NSCLC 3 | MP-152 | ADC | G3 | 59 | Male |
| NSCLC 4 | MP-153 | SCC | G3 | 49 | Female |
| NSCLC 5 | MP-155 | ADC | G2 | 75 | Male |

## Supplementary Table S4: List of subject analyzed in Western Blot studies.

| **Nr.** | **Patient** | **ADC/SCC** | **Grading** | **Age** | **Gender** |
| --- | --- | --- | --- | --- | --- |
| P1 | MP-107 | ADC | G1 | 54 | Female |
| P2 | MP-118 | ADC | G1 | 67 | Female |
| P3 | MP-77 | ADC | G2 | 64 | Female |
| P4 | MP-94 | ADC | G2 | 77 | Female |
| P5 | MP-15 | ADC | G3 | 63 | Male |
| P6 | MP-81 | ADC | G3 | 61 | Male |
| P7 | MP-21 | SCC | G1 | 41 | Male |
| P8 | MP-23.2 | ADC | G2 | 73 | Male |
| P9 | MP-34.1 | ADC | G2 | 51 | Female |
| P10 | MP-98 | ADC | G3 | 55 | Female |
| P11 | MP-99 | ADC | G3 | 62 | Male |
| P12 | MP-68 | ADC | G3 | 42 | Male |
| P13 | MP-78 | ADC | G3 | 80 | Female |
| P14 | MP-80 | ADC | G3 | 62 | Male |
| P15 | MP-83 | ADC | G3 | 60 | Female |
| P16 | MP-89 | ADC | G3 | 61 | Female |
| P17 | MP-93 | ADC | G3 | 66 | Male |
| P18 | MP-11 | Pneumonia | | 51 | Male |
| P19 | MP-26 | ADC | G3 | 52 | Female |
| P20 | MP-6 | Metastatic patient | G3 | 79 | Female |
| P21 | MP-18 | Metastatic patient | G2 | 52 | Female |
| P22 | MP-62 | ADC | G2 | 80 | Female |
| P23 | MP-59 | ADC | G2 | 71 | Male |
| P24 | MP-56 | ADC | G2 | 68 | Female |
| P25 | MP-55 | ADC | G3 | 64 | Female |
| P26 | MP-51 | ADC | G3 | 61 | Male |
| CN-1 | CN-6 | Control | | 53 | Male |
| CN-2 | CN-9 |  |  | ? | ? |
| CN-3 | CN-2 |  |  | 61 | Male |

## Supplementary Table S5: List of antibodies used for immunohistochemistry (IHC).

| Antigen | Clone | Dilution | Supplier |
| --- | --- | --- | --- |
| CD3 | PS1 | 1:50 | Abcam |
| Foxp3 | 236A/E7 | 1:50 | Thermo Fisher Scientiﬁc |
| IL-9 | Polyclonal | 1:100 | Abcam, Cambridge, England |
| IL-9R | F-3 | 1:100 | Santa Cruz Biotechnology, Inc., Texas, USA |

## Supplementary Table S6: List of antibodies used for Flow Cytometry.

| Reactivity | Antigen | Fluorochrome | Clone | Supplier |
| --- | --- | --- | --- | --- |
| Mouse | CD4 | BV421 | RM4-5 | BD Bioscience, Franklin Lakes, USA |
|  | CD4 | Alexa488 | RM4-5 | BD Bioscience |
|  | CD4 | PerCPCy5.5 | RM4-5 | BD Bioscience |
|  | CD8 | FITC | 53-6.7 | BD Bioscience |
|  | CD8 | PerCP | 53-6.7 | Biolegend, Fell, Germany |
|  | CD25 | PerCPCy5.5 | PC61 | BD Bioscience |
|  | CD25 | PE/Cy7 | PC61 | Biolegend |
|  | Foxp3 | APC | 3G3 | Miltenyi Biotec, Bergisch Gladbach, Germany |
|  | Foxp3 | PE | FJK-16s | eBioscience |
|  | pSTAT5 | PE | 47/Stat5 | BD Bioscience |
|  | Tbet | PE | O4-46 | BD Bioscience |
|  | TNFα | FITC | MP6-XT22 | BD Bioscience |
|  | IL-9 | PE | RM9A4 | Biolegend |
| Human | CD3 | FITC | UCHT1 | BD Bioscience |
|  | CD3 | APC-Fire750 | UCHT1 | Biolegend |
|  | CD4 | FITC | OKT4 | eBiosicence |
|  | CD25 | PE-Cy7 | BC96 | eBioscience |
|  | CD95 (FAS) | PE-Cy5 | DX2 | BD Pharmingen |
|  | CD119 | PE-vio770 | REA161 | Miltenyi Biotec |
|  | CD129 | PE | AH9R7 | Biolegend |
|  | EpCAM | APC | 9C4 | Biolegend |
|  | Foxp3 | Alexa647 | 259D/C7 | BD Bioscience |

## Supplementary Table S7: List of primers used for Quantitative Real-Time PCR.

| Species | Gene | Primer Sequence |
| --- | --- | --- |
| Human | *FOXP3* | For: 5´-AAC AGC ACA TTC CCA GAG TTC CT-3`  Rev: 5´-GGT CCT TTT CAC CAG CAA GCT-3` |
|  | *HPRT* | For: 5′-TGA CAC TGG CAA AAC AAT GCA-3`  Rev: 5′-GGT CCT TTT CAC CAG CAA GCT-3` |
|  | *IL2* | For: 5´-AAG AAG GCC ACA GAA CTG AAA C-3`  Rev: 5´-ATG GTT GCT GTC TCA TCA GC-3` |
|  | *TNFα* | For: 5´- CCC TGA AAA CAA CCC TCA GA-3´  Rev: 5´- AAG AGG CTG AGG AAC AAG CA-3´ |
| Mouse | *Hprt* | For: 5´-GCC CCA AAA TGG TTA AGG TT-3`  Rev: 5′-TTG CGC TCA TCT TAG GCT TT-3` |
|  | *Il9r* | For: 5´-TGC CCA GGA GAC ACA TCA AG-3`  Rev: 5´-TAC CCC AGG TCA GGA CAC AA-3 |
|  | *Spi1* | For: 5´-GCA TCT GGT GGG TGG ACA A-3`  Rev: 5`-TCT TGC CGT AGT TGC GCA G-3` |

## Supplementary Table S8: List of antibodies used for Western Blot.

| Antigen | Clone | Dilution | Supplier | Catalog number |
| --- | --- | --- | --- | --- |
| GAPDH | Rabbit | 1:1000 | Cell Signaling | Cat #2118 |
| IL-9 | Rabbit | 1:1000 | Abcam | Cat #ab11915 |

| Antibody | Dilution | Company | Catalog number |
| --- | --- | --- | --- |
| Anti-rabbit HRP- linked antibody | 1:2000 | Cell Signaling | Cat #7074S |
| Anti-mouse HRP- linked antibody | 1:2000 | Cell Signaling | Cat #7076S |

# Supplementary Figures


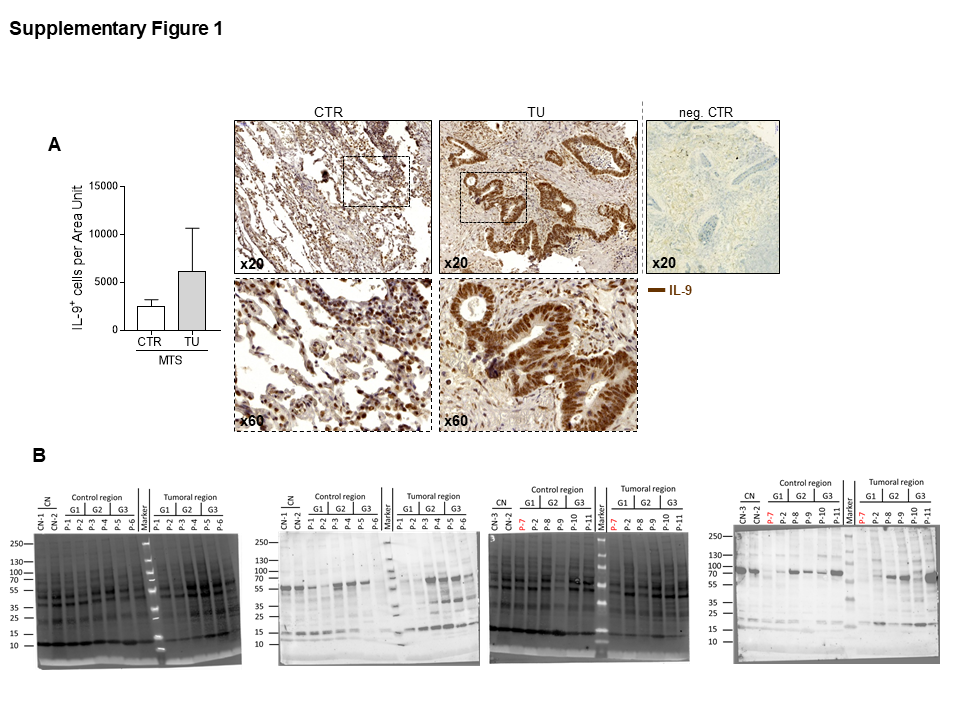


**Supplementary Figure 1.** **Induction of IL-9 in the tumoral region of the lung of Patients with NSCLC** **(A)** Pictures were imported in Power point from the scanned sections immunostained with anti IL-9 antibodies via IHC. **(B)**.Uncut western blots immunoblotted for IL-9.


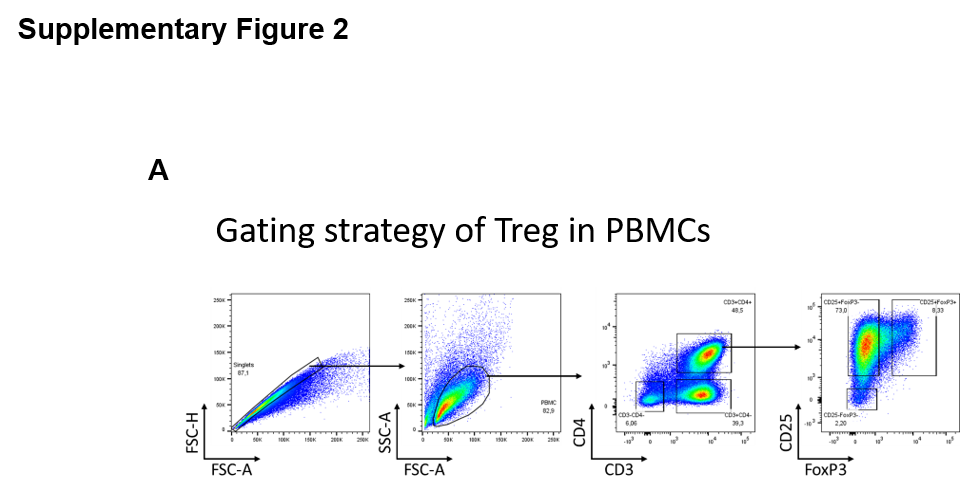


**Supplementary Figure 2. Gating strategy of Treg in PBMCs from patients with NSCLC and healthy controls.**


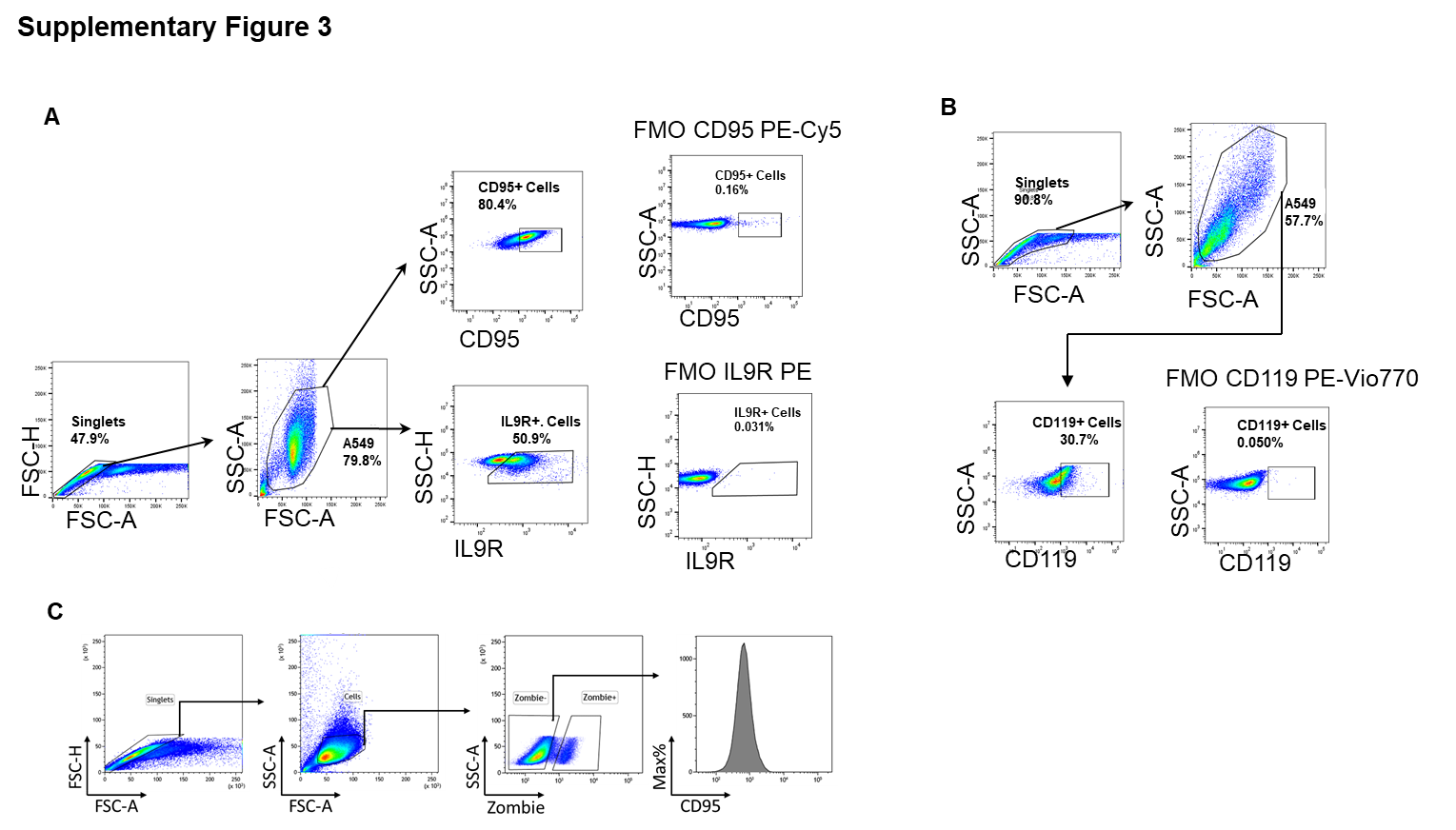


**Supplementary Figure 3. Gating strategies of A549 cells. (A)** Gating strategies for IL9R and CD95 expression on A549, related to Figure 4 A, B and Figure 5 A. **(B)** Gating strategy for IFNɣ receptor (CD119) expression on A549, related to Figure 4 C. **(C)** Gating strategy for the CD95 expression on A549, related to Figure 5 B, C.


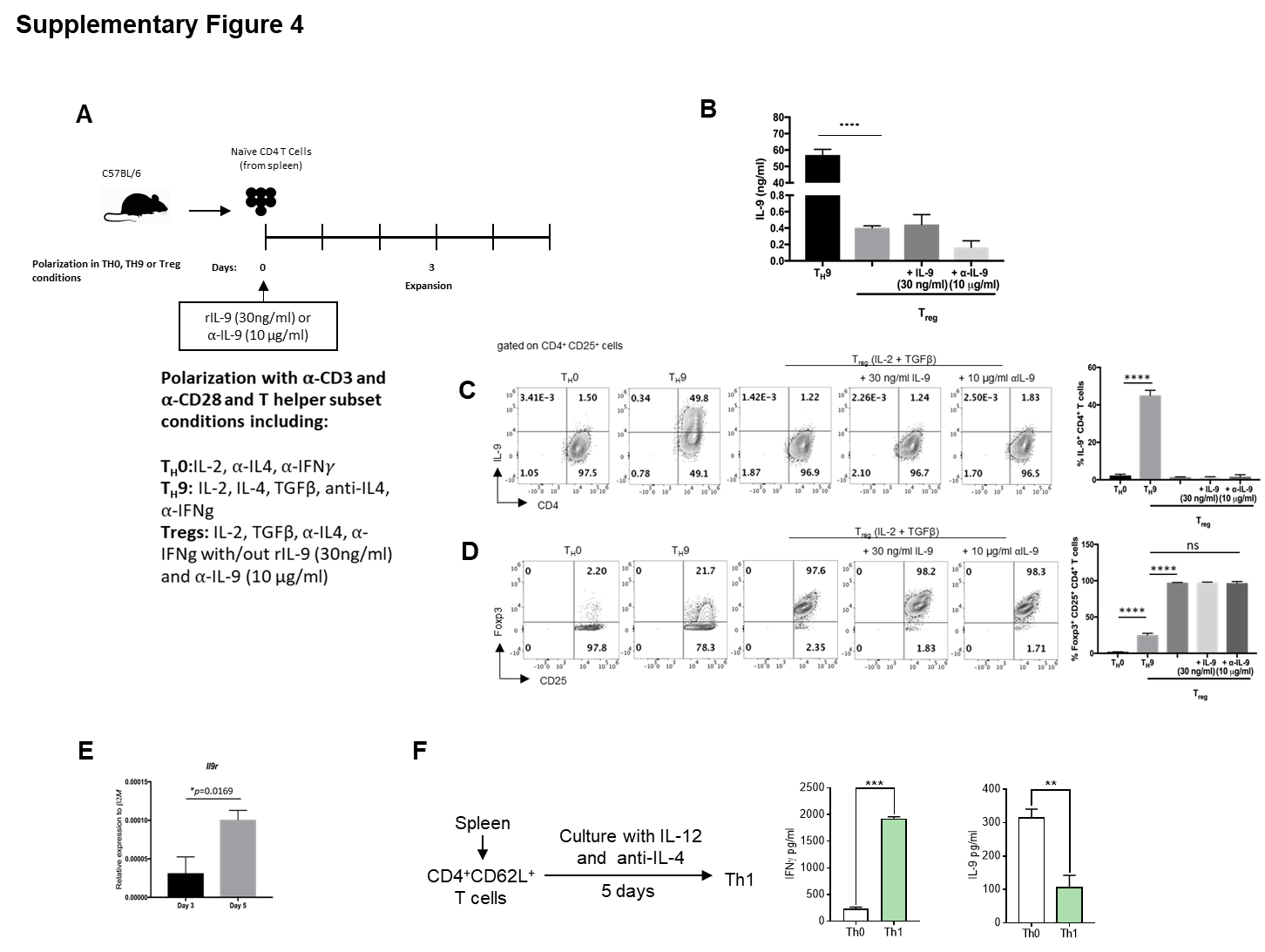


**Supplementary Figure 4. Induced *IL-9R mRNA* expression at late stage of Treg differentiation. (A)** Experimental design of Th9 and T reg cell differentiation. **(B)** ELISA analysis of IL-9 (pg/ml) in supernatant obtained from *in vitro* differentiated Naïve CD4^+^ T cells from wild-type mice were cultured *in vitro* for 5 days under Th0, Th9 and Treg polarization conditions with or without IL-9 (30 ng/ml) or ⍺-IL-9 antibodies (10 μg/ml). IL-9 secretion measured via ELISA in T_H_9 and T_reg_ subsets differentiation with/out IL-9 or ⍺-IL-9 (N_Th9_=3; N_Treg_=3). **(C, D)** Representative dot-plots showing IL-9 and Foxp3 expression measured in Th0, Th9 and Treg subsets differentiated with/out IL-9 or ⍺-IL-9 using intracellular staining followed by flow-cytometric analysis. Cells were gated on viability followed by CD4^+^ CD25^+^ populations. **(E)** IL-9 receptor expression measured on day 3 and day 5 of Treg differentiation *in vitro* (N_d3_=3; N_d5_=3). N values are given per group. Tukey's multiple comparisons test was used for statistical analysis for bar graphs. (*p<0.05, **p<0.01, ***p<0.001, ****p<0.0001). Bar charts indicate mean values ± SEM. **(F)** Experimental design for the *in vitro* differentiation of naïve CD4^+^CD62L^+^ splenic T cells towards Th1 (5 µg/ml anti-IL4, 12 ng/ml IL-12) phenotype. ELISA analysis of IFNγ (pg/ml) (left) and IL-9 (pg/ml) (right) in supernatant obtained from *in vitro* differentiated Th1 cells (N_Th0_=4; N_Th1_=4). Statistical analysis was done using using student´s two-tailed t-test *P,0.05; **P,0.01; ***P,0.001.
